# Semiconducting Polymer Nanoparticles as Multimodal Agents for Optical and Magnetic Resonance Imaging

**DOI:** 10.1002/adhm.202500195

**Published:** 2025-06-26

**Authors:** Faysal A. Farah, Yu Qin, Mark A. Green, James D. E. T. Wilton‐Ely

**Affiliations:** ^1^ Department of Chemistry Imperial College London Molecular Sciences Research Hub White City Campus London W12 0BZ UK; ^2^ Department of Physics King's College London Strand London WC2R 2LS UK

**Keywords:** magnetic resonance imaging, multimodal imaging, optical imaging, semiconducting polymer nanoparticles

## Abstract

Semiconducting polymer nanoparticles (SPNs) are a very promising class of fluorescent nanoparticles that exhibit many advantageous optical and biological properties. However, their potential for optical imaging in clinical applications is currently restricted by limited tissue penetration. Multimodal SPN‐based contrast agents that integrate several imaging modalities into one platform are able to yield a wealth of information through the use of different imaging mechanisms. The inclusion of magnetic resonance imaging (MRI), with its good spatial resolution and deep tissue penetration, enables SPNs to combine the complementary advantages of MRI and optical imaging. This short review explores the approaches adopted in the literature in the nascent field of multimodal optical‐MRI SPN‐based probes.

## Graphical abstract

Semiconducting polymer nanoparticles (SPNs) exhibit many advantageous optical and biological properties. Multimodal SPN‐based contrast agents that integrate several imaging modalities yield a wealth of information through the use of different imaging mechanisms. This review covers the emerging area of SPNs that combine exceptional brightness and excellent photostability with the spatial resolution and deep‐tissue imaging offered by magnetic resonance imaging (MRI).

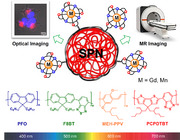

## 1 Introduction

**Figure 1:**
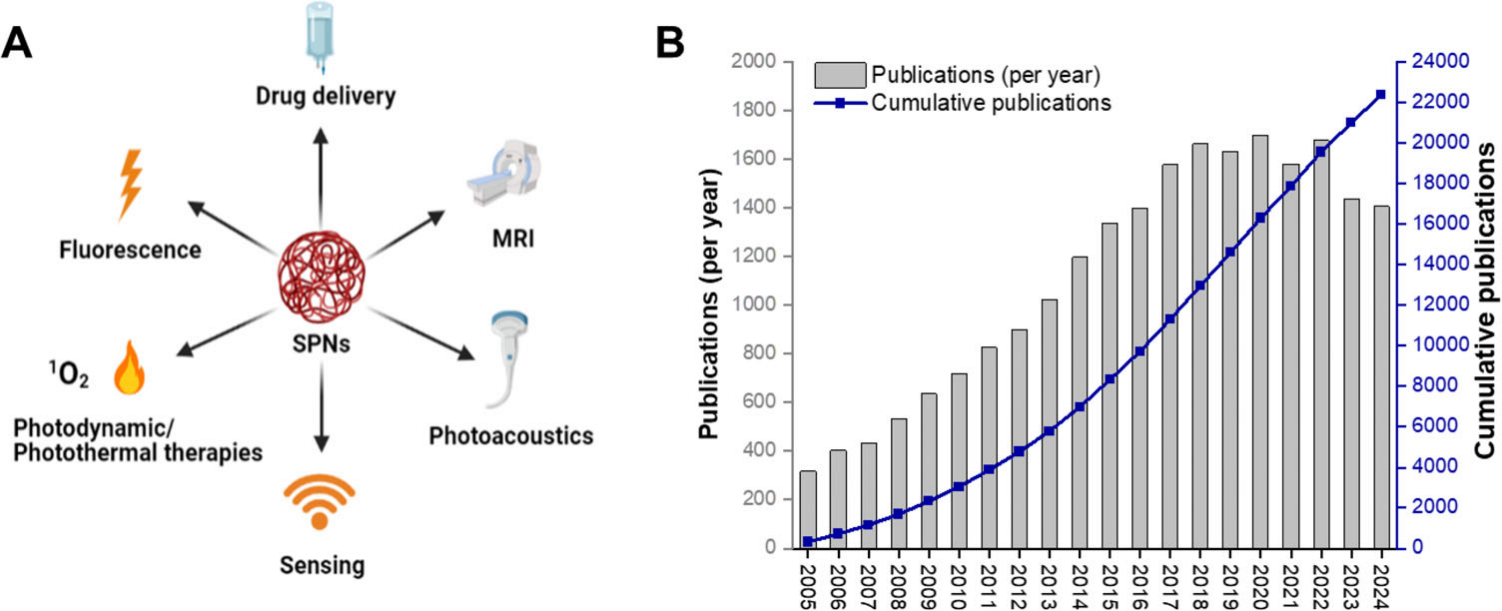
A) A summary of the medical applications of SPNs; B) Number of papers that have made use of SPNs published per year and total accumulated publications over time from 2005 to 2024 using the following search criteria: conjugated polymer nanopart^*^ or semiconducting polymer nanopart^*^ or SPN or CPN.

Semiconducting polymer nanoparticles (SPNs) are versatile nanomaterials that have attracted both academic and industrial interest as fluorescent nanoparticles for use in biomedical applications.^[^ ^1^, ^2^, ^3^, ^4^, ^5^, ^6^, ^7^ ^]^ Derived from the self‐assembly of conjugated polymers (CPs), these fascinating materials exhibit highly desirable optical properties such as a large absorption cross‐section and exceptional brightness per particle (defined as the product of quantum yield and absorption cross‐section) when compared to other nanoparticle‐based fluorescent probes.^[^ ^8^ ^]^ In addition to their exceptional brightness, SPNs also display fast radiative rates, excellent photostability, and minimal blinking behavior making them suitable for a wide variety of applications including cellular labeling studies, sensing, and in vivo imaging (**Figure** **1**).^[^ ^9^ ^]^

In recent years, the development of fluorescent probes designed to operate in the near‐infrared region (NIR) has become the subject of intense investigation owing to minimal scattering and absorption of NIR light by biological tissue.^[^ ^10^ ^]^ These studies usually focus on either modulating the emission spectra of SPNs through a rational donor‐acceptor design of the CP,^[^ ^11^ ^]^ doping with a NIR emitting dye,^[^ ^12^ ^]^ or by employing time‐resolved luminescence imaging that exploits the very long fluorescence lifetimes of NIR‐emitting SPNs.^[^ ^13^ ^]^ However, despite improvements to the synthesis and preparation of SPNs for use in the NIR‐I/II region (700–1700 nm), the significant cost of the instrumentation required to image in the NIR window, and the associated modest enhancement in penetration depth, currently hinders their use in a clinical setting. An alternative approach to address the issues surrounding tissue attenuation is to incorporate an additional imaging modality to complement the strengths of optical imaging.^[^ ^14^ ^]^ A variety of imaging modalities exist that can be coupled with optical imaging and are classified according to their contrast mechanism: photoacoustic imaging (PAI), magnetic resonance imaging (MRI), positron emission tomography (PET), single‐photon emission computed tomography (SPECT), X‐ray computed tomography (CT) and ultrasound (US).^[^ ^15^ ^]^ Of these techniques, MRI offers many attributes that complement fluorescence imaging, such as its non‐ionizing nature and its capacity to yield images with high spatial resolution unrestricted by depth within the body. Furthermore, recent studies have demonstrated that ratiometric MRI probes can deliver both molecular insights into pathological processes and detailed anatomical information, all non‐invasively. Importantly, these probes minimize the impact of fluctuations in probe concentration, thereby potentially addressing a key limitation of fluorescence imaging, which is often affected by concentration dependency.^[^ ^16^ ^]^ The great potential offered by the combination of these two imaging modalities for the generation of complementary data has driven research in this field and it is now clear that SPNs provide an ideal platform to develop dual‐mode optical‐MRI contrast agents.^[^ ^17^ ^]^

Integrating optical imaging with MRI through the use of a multimodal imaging platform has been recognized as a powerful approach for both clinical applications and biomedical research, as this combination leverages the strengths of each modality.^[^ ^14^ ^]^ MRI delivers high‐resolution anatomical and functional data, such as brain structure, blood flow, and tissue composition, while optical imaging techniques, including fluorescence imaging, provide molecular and cellular‐level insights, particularly valuable in preclinical studies involving gene expression and protein activity.

Similarly, by combining MRI and optical imaging, researchers and clinicians can achieve a more comprehensive understanding of biological processes. Indeed, this integration enhances both sensitivity and specificity in detecting molecular changes (optical imaging) with high spatial resolution and soft tissue contrast (MRI). This synergy contributes to improved diagnostic precision and holds promise for the earlier detection of disease. Furthermore, the dual‐modality approach enables real‐time functional and molecular imaging. Optical imaging can monitor dynamic biological events, such as calcium signaling and metabolic activity, while MRI can concurrently capture functional changes, like those observed in functional MRI (fMRI), allowing for the correlation of molecular activity with physiological outcomes.^[^ ^18^ ^]^

The combined use of these modalities is also highly beneficial in drug development and pharmacological research.^[^ ^19^ ^]^ In preclinical settings, optical imaging can trace drug distribution and target engagement, while MRI can evaluate tissue‐level responses and structural alterations over time, offering a more holistic assessment of therapeutic efficacy.

Finally, there is growing interest in fluorescence‐guided surgery.^[^ ^20^ ^]^ Systems that integrate optical and MR imaging can delineate tumor margins or identify specific cell populations using fluorescent markers, while MRI provides precise, non‐invasive anatomical localization prior to surgical intervention. This integration enhances surgical planning and execution, potentially improving patient outcomes.

The growing interest in SPNs has led to several reviews on the general topic of multimodal contrast agents based on SPNs.^[^ ^2^, ^5^, ^21^ ^]^ However, the use of SPNs as dual MRI‐optical probes has not been addressed in detail previously. Against the backdrop of a rapidly growing number of papers being published in this field (Figure 1B), this review combines a summary of key work on SPNs with an emphasis on design strategies for optimizing their utility in combined optical‐MR imaging. The approaches recently adopted in the development of dual‐modal optical‐MRI SPN‐based probes (**Table** **1**) will be outlined, highlighting the aspects that will aid the design of the next generation of probes. Due to the extensive existing literature on SPNs, optical imaging, and MRI, this review will focus solely on multimodal SPNs for optical‐MR imaging. Examples of SPNs derived from low‐bandgap CPs, more commonly used in photoacoustic imaging (PAI), such as polypyrrole (PPy), will also be covered to provide relevant background. To provide context and a useful summary for the reader, a brief outline of the basic principles of optical imaging and MRI and the parameters controlling the efficiency of contrast agents will be presented following an overview of the advantages of SPNs for optical imaging.

**Table 1:** Examples of semiconducting polymer nanoparticles (SPNs) used as contrast agents for magnetic resonance imaging are mentioned in this review.

CP	Size [nm]	SPION Size [nm]	Photophysical properties	*r_1_*	*r_2_*	Field [T]	Observations from in vivo results	Ref
				(mM^−1^·s^−1^)				
MEH‐PPV	≈114	‐	λ_abs_ = 495 nm λ_em_ = 592 nm QY_Gd‐SPNs_ = 1.5 QY_polymer_ = 15.9 (in DCM)	20.8	‐	3.0	Model: Mice (subcutaneous, intramuscular, and deep chest cavity injections). Imaging performance: Strong fluorescence was visible after subcutaneous and intramuscular injection. No detectable fluorescence was observed following the deep chest cavity injection.	[74]
HE‐CP_10_	100	‐	λ_abs_ = 300, 380, 520 nm λ_em_ = 650 nm	105.4	‐	1.5	Model: Mice bearing human breast tumors (intravenous injection). Imaging performance: Achieved highest *T_1_*‐weighted MRI signal at 330 min post‐injection at 0.5 T.	[75]
PCPDTBT	112	‐	λ_abs_ = 675 nm	75.0	‐	9.4	Model: Mice bearing HepG2 tumor (intratumoral injection). Imaging performance: Significant MRI and PAI signal enhancement observed in tumors.	[80]
PPy	69	‐	λ_abs_ = 808 nm	10.6	‐	3.0	Model: Mice‐bearing glioma U87‐MG tumor (intratumoral injection). Imaging performance: Significant increases in both MRI and PAI signals detected in tumors.	[79]
Polyfluorene	110	‐	λ_abs_ = 377 nm λ_em_ = 418, 440 nm	13.2	‐	0.5	No in vivo data reported	[88]
PFP	60	‐	λ_abs_ = 352 nm λ_em_ = 450 nm	0.3	‐	3.0	Model: Mice bearing A549 tumor (intratumoral injection). Imaging performance: Significant increase in *T_1_*‐weighted MRI signal at the tumor site after injection.	[91]
MEH‐PPV	111 ‐ 643	6.5±3.0	λ_abs_ = 500 nm, λ_em_ = 600 nm QY = 2.2%	‐	152.0 (*R_2_* value)	3.0	No in vivo data reported	[103]
PCPDTBT	176.0	‐	λ_abs_ = 516 nm λ_em_ = 636 nm	‐	53.3	7.0	Model: Mice bearing hepatoma tumor (intravenous injection). Imaging performance: Tumors appeared 2× brighter by fluorescence imaging, *T_2_* relaxivity value of 53.3 mM⁻¹·s⁻¹ at 7.0 T measured 5 h post‐injection.	[105]
CN‐PPV	≈30	2.2	λ_em_ = 575 nm QY = 0.6	6.6	27.9	7.0	Model: Mice bearing GL261 glioma (intravenous injection). Imaging performance: Significant *T_2_*‐weighted MRI signal decrease at the tumor site.	[106]
PCPDTBT	147	5.0	λ_abs_ = 780 nm	‐	309.3	3.0	No in vivo data reported	[110]
PPy	≈100	6.0	No data	‐	72.1	3.0	Model: Mice bearing 4T1 breast tumors (intravenous injection). Imaging performance: Strong tumor uptake and accumulation were detected by both MR and PAI, *T_2_*‐weighted MR contrast showed a 75% signal decrease at 24 h post‐injection, and a strong PAI signal was observed in tumors.	[114]
PFBT‐C50/PFTBT	23	2.2	λ_abs_ = 470 nm λ_em_ = 660 nm	7.0	26.8	7.0	Model: Mice bearing A549 lung cancer tumors (intravenous tail vein injection). Imaging performance: In NIR fluorescence imaging, rapid whole‐body signal was obtained at 1 h; tumor‐specific high signal and clear delineation at 6 h post‐injection; MRI showed significant tumor enhancement in both *T_1_*‐ and *T_2_*‐weighted images.	[107]
PDPP3T	165	7.5	λ_abs_ = 750 nm The major peak of PA is 750 nm	‐	98	7.0	Model: Mice bearing 4T1 tumors (intravenous injection). Imaging performance: Achieved clear PAI and *T₂*‐weighted MRI visualization of tumors; tumor accumulation of CP‐IO produced a strong PAI signal and distinct tumor margin, peaking at 7 h post‐injection; MRI showed corresponding tumor signal enhancement with signals persisting in the tumor region for at least 24 h, enabling prolonged multimodal imaging.	[109]
PPy	150	8–10	λ_abs_ = 700–1100 nm	‐	87.0	3.0	Model: Balb/c mice with subcutaneous 4T1 cancer tumors (single intratumoral injection). Imaging performance: *T₂*‐weighted MRI showed strong contrast at tumor sites.	[113]
PPy	82.7	≈11.9	λ_abs_ = 700–900 nm	‐	127.2	7.0	Model: BALB/c mice bearing subcutaneous 4T1 cancer tumors (intravenous). Imaging performance: *T₂*‐weighted MRI showed clear tumor accumulation and strong contrast at 4 h post‐injection, which persisted to 24–48 h; in PAI, tumor signal peaked at 4–8 h post‐injection.	[115]
PPy	≈15	‐	λ_abs_ = 1000 ‐ 1700 nm	‐	0.623^a)^	0.5	Model: BALB/c nude mice (intravenous injection). Imaging performance: In MRI, *T₂*‐weighted imaging showed clear darkening in the liver at 2 h post‐injection (weaker than Fe₃O₄ NPs) and remained MRI‐active for at least 5 h; in PAI, strong photoacoustic signal detected in the liver at 1280 nm, enabling 3D visualization of liver tissue; comparison made to standard.	[117]

^a))^ Relaxivity normalized to the concentration of polarons measured in the solid state

## 2 An Introduction to SPNs

The inherent hydrophobicity of the polymer backbone in CPs allows them to be used to form semiconducting polymer nanoparticles (SPNs) that can be dispersed in aqueous media.^[^ ^22^ ^]^ SPNs are soft materials consisting of weakly bound polymers that adopt a spherical shape in order to minimize the interfacial energy between the hydrophobic core and the surrounding aqueous environment.^[^ ^9^ ^]^ By definition, SPNs consist of CPs with a volume fraction or weight concentration of at least 50% (ideally >80%), which excludes other types of nanomaterials into which the CPs have been doped.^[^ ^23^ ^]^

### 2.1 SPNs Preparation Strategy

**Figure 2:**
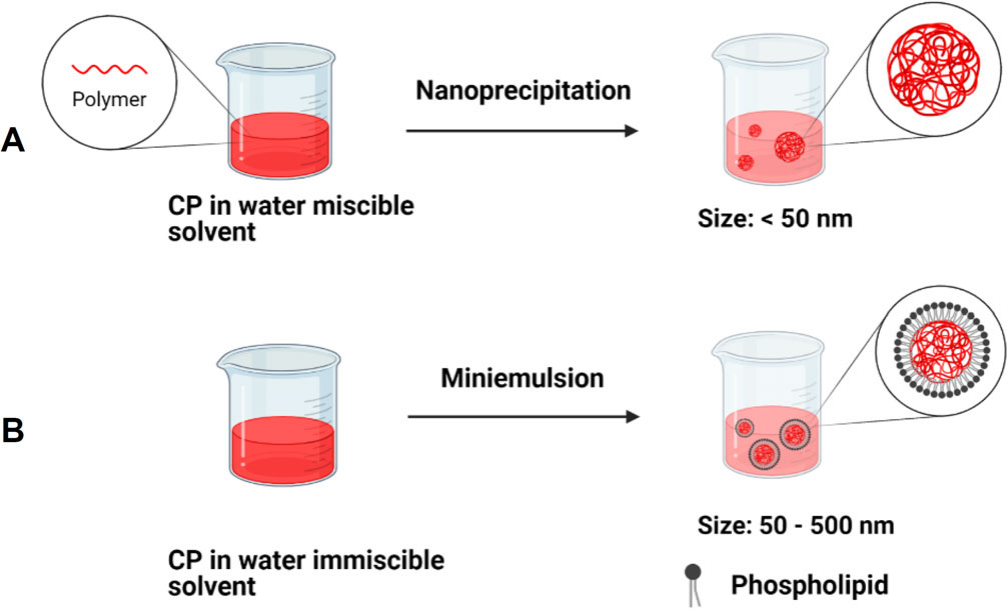
The two most common post‐polymerization preparation techniques for making SPNs are the A) nanoprecipitation and B) miniemulsion methods.

Several techniques have been developed to generate SPNs that are either based directly on the monomer units themselves (e.g., emulsion polymerization techniques)^[^ ^24^ ^]^ or that commence from a pre‐prepared polymer (e.g., post‐polymerization).^[^ ^22^ ^]^ The vast majority of SPNs are made up of several polymer chains with the exact number depending upon the molecular weight of the conjugated polymer and the size of the resulting SPN.^[^ ^22^ ^]^ The choice of preparative route is often dictated by the application being addressed and, for biomedical applications, the latter approach is often favored for its ease of preparation and the availability of a large library of existing CPs.^[^ ^8^ ^]^ Post‐polymerization dispersion of CPs can be achieved through miniemulsion and nanoprecipitation routes (**Figure** **2**).

The earliest reports of SPNs date back to the 1980s and involve the in situ emulsion polymerization of electrically conducting conjugated polymers (e.g., polyacetylene).^[^ ^25^, ^26^ ^]^ Since then, nanoparticles derived from other classes of conjugated polymers with various size regimes have been developed for use in biological applications.^[^ ^27^ ^]^ A variation of this approach, termed passive miniemulsion, utilizes a pre‐prepared polymer to generate SPNs in a similar manner. This method proceeds via the generation of a heterophasic system compromised of small droplets of the CP dissolved in a good solvent dispersed in an antisolvent with respect to the CP.^[^ ^28^ ^]^ On application of a shear force, sub‐micrometre droplets are formed, which are typically stabilized by surfactants to prevent aggregation.^[^ ^1^ ^]^ In some cases, a hydrophobe is added alongside the surfactants to provide further stabilization with respect to Ostwald ripening. Subsequent removal of the polymer solvent(s) generates a highly stable dispersion of SPNs with particle sizes ranging from 20 to 500 nm, which exhibit long‐term colloidal stability.^[^ ^29^ ^]^

**Figure 3:**
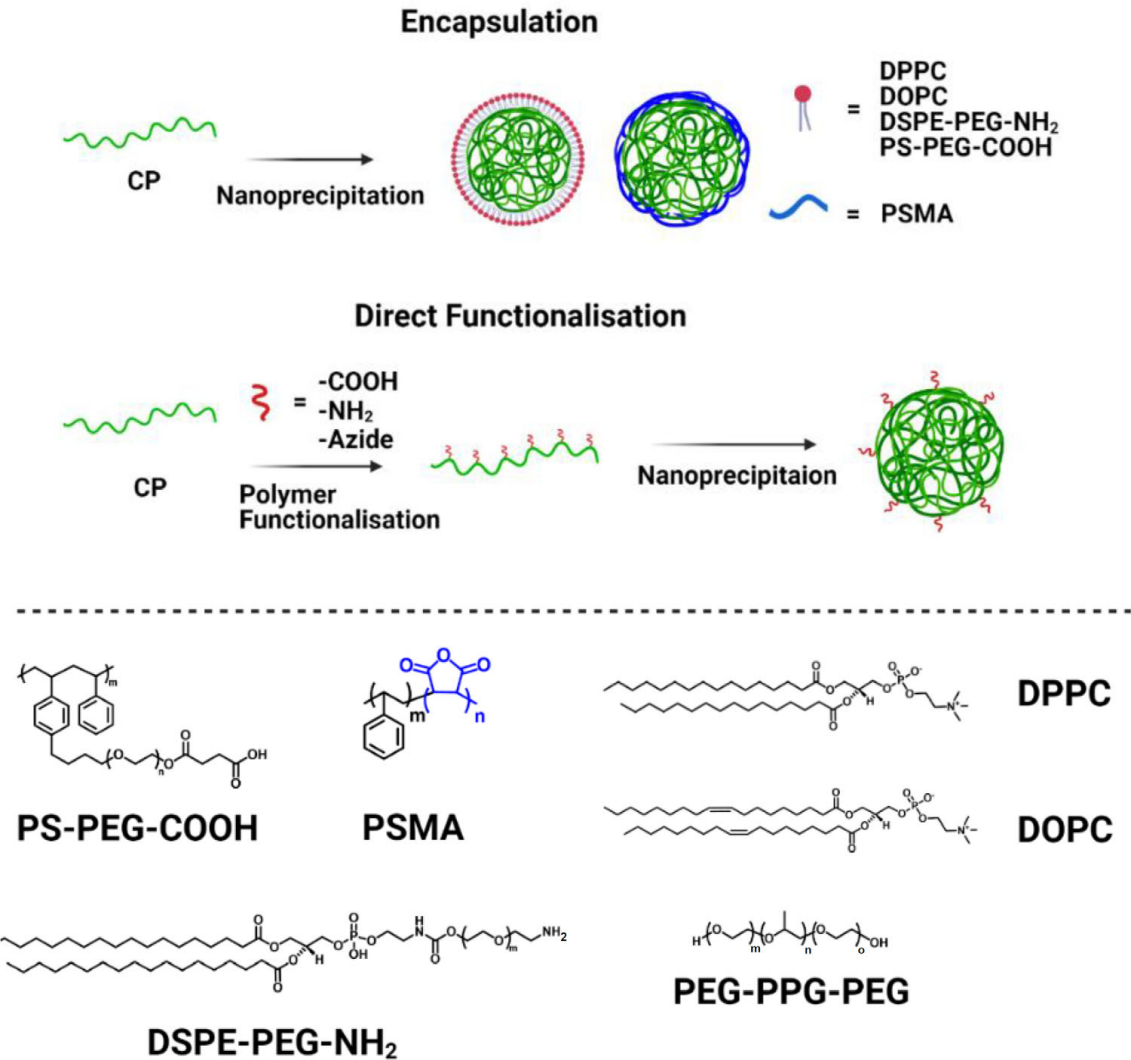
Surface functionalization of SPNs through encapsulation of SPNs with either phospholipids or amphiphilic copolymers. Chemical structures are shown of polystyrene‐polyethylene glycol‐carboxylic acid (PS‐PEG‐COOH), PSMA, 1,2‐dipalmitoyl‐sn‐glycero‐3‐phosphocholine (DPPC), 1,2‐dioleoyl‐sn‐glycero‐3‐phosphocholine (DOPC), 1,2‐distearoyl‐sn‐glycero‐3‐phosphoethanolamine‐N‐[amino(polyethylene glycol)] (DSPE‐PEG‐NH_2_) and poly(ethylene glycol)‐block‐poly(propylene glycol)‐block‐poly(ethylene glycol) (PEG‐PPG‐PEG).

The nanoprecipitation (or reprecipitation) route exploits the hydrophobic nature of the polymer backbone to induce chain collapse following a change in solvent quality.^[^ ^4^ ^]^ Nanoprecipitation was first employed with electrically‐conducting conjugated polymers by Kasai and co‐workers and has since been adapted by McNeill and co‐workers to produce SPNs with smaller size regimes for biological applications, typically of ≈5–80 nm.^[^ ^7^, ^30^ ^]^ In this technique, the hydrophobic CP is dissolved in a water‐miscible organic solvent before the polymer solution is rapidly transferred to an excess of water (**Figure** **3**). This sudden reduction in solvent quality encourages the formation of nucleating units consisting of several polymer units that subsequently grow in size via a diffusion‐limited process. This process occurs until further addition of polymer units becomes increasingly difficult and the nanoparticles become “kinetically locked.” The final stage in the formation of SPNs via the nanoprecipitation method is characterized by an extremely slow change in size due to the exchange of polymer units in order to reach equilibrium.^[^ ^31^ ^]^ Interestingly, in some cases, researchers have observed that SPNs derived from the nanoprecipitation method showed remarkable colloidal stability in the absence of surfactant molecules, showing no sign of aggregation even after several weeks.

The choice of preparation method can affect the size and internal structure of the SPNs, which in turn can influence the imaging properties. However, some of these properties can be adjusted for within each method, allowing similar optical properties and sizes to be achieved overall. Thus, both methods can be used to achieve desirable imaging capabilities. Further details can be found in a recent review on the topic.^[^ ^32^ ^]^

### 2.2 Optical Imaging and Fluorescence Properties of SPNs

Optical imaging provides a wealth of information on cellular structure, function, and pathways, as well as producing highly detailed images of organs and tissues by exploiting the properties of light such as absorption, emission, scattering polarisation, coherence, and reflectance.^[^ ^33^ ^]^ Most commonly, fluorescence properties are utilized, which involves the absorption of light with a specific wavelength followed by the emission of light at a different wavelength.^[^ ^34^ ^]^ Much of the popularity of fluorescence imaging stems from its ability to study biological species and processes at the subcellar length scale in a non‐destructive manner. Additionally, multiple properties of fluorescence can be measured such as intensity, wavelength, lifetime, and polarization, which enable complex biological samples to be studied through the lens of multiplex imaging.

Fluorophores are a class of optically active compounds that undergo fluorescence following an excitation event. The majority of the fluorophores used in fluorescence imaging are synthetic compounds that contain a high degree of *π*‐conjugation, such as conjugated polymers.^[^ ^35^ ^]^ For example, indocyanine green (ICG) is an FDA‐approved dye used in surgical medicine that belongs to a family of small‐molecule fluorescent cyanine dyes with an extended *π*‐conjugated system.^[^ ^36^ ^]^ Further examples of molecular dyes with extended *π*‐conjugated systems include rhodamine B, fluorescein, and boron dipyrromethene (BODIPY). However, despite showing promise as fluorescent dyes in cell imaging studies, such organic fluorophores suffer from drawbacks such as photobleaching, which causes the fluorescence signal to fade permanently.^[^ ^37^ ^]^ Other phenomena, such as rapid blinking, poor brightness (defined as the product of quantum yield and absorption coefficient constant), and a small Stokes shift further prevent their use at low concentrations and can hamper their clinical translation.^[^ ^3^, ^37^ ^]^ In addition to the optical properties of the fluorophore, it is also important to consider their physicochemical properties such as its biocompatibility, inertness, size, solubility, and its chemical stability.

**Figure 4:**
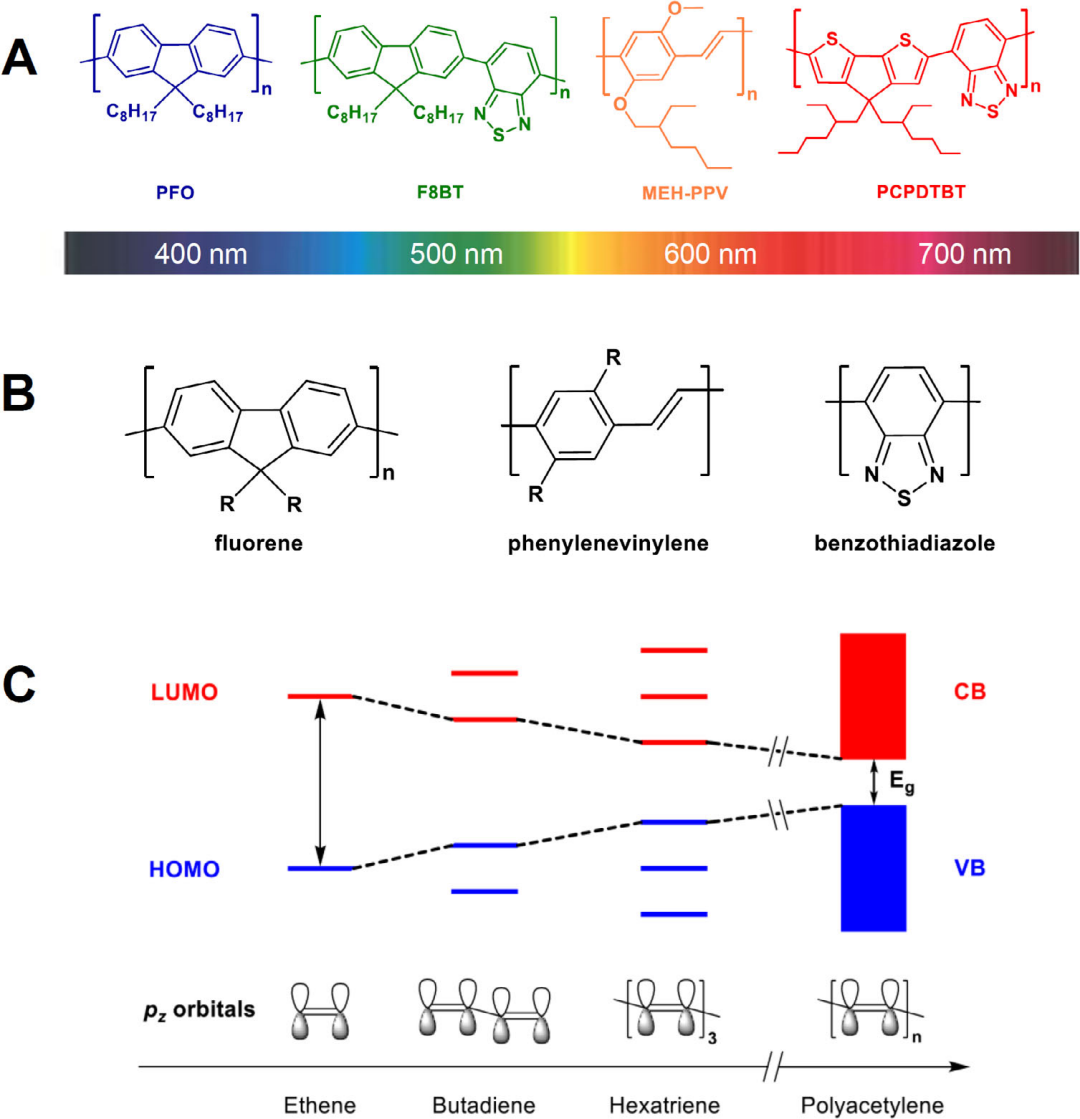
A) Chemical structures of selected conjugated polymers with their corresponding emission regions in the visible light spectrum; B) Chemical structures of common monomer units used in CPs; C) Representation of the band structure with polyacetylene as an example with the band structure formed through sequential addition of ethene units resulting in a reduction in the bandgap (monomeric, dimeric, trimeric and polymeric energy levels are also shown, CB = conduction band, VB = valence band).

Conjugated polymers (CPs) are a more recent class of fluorophores that have attracted both academic and industrial interest as organic semiconductors owing to their favorable optoelectronic properties, synthetic versatility, and ease of processability.^[^ ^38^ ^]^ This has led their widespread use in devices such as organic light‐emitting diodes (OLEDs), polymer solar cells (PSCs), organic field‐effect transistors (OFETs), and many other applications.^[^ ^39^ ^]^ More importantly, in the context of this review, they exhibit remarkable brightness, minimal blinking, and reduced photobleaching, which give them great potential as probes for fluorescence imaging.^[^ ^40^ ^]^ Several distinct classes of CPs have been developed, which, along with their derivatives, have emission colors that span the entire visible light spectrum (**Figure** **4**A). Polymers derived from fluorene, phenylenevinylene, and benzothiadiazole (Figure 4B) are prominent in the field, along with mixed systems that combine an electron‐deficient monomer and an electron‐rich monomer, often referred to as donor‐acceptor polymers.

A characteristic feature of CPs is a planar polymer backbone comprised of a system of alternating single and double (or triple) bonds. When two adjacent pz orbitals of the same symmetry overlap, as is the case in CPs, two new molecular orbitals are formed leading to a higher energy anti‐bonding (*π* ^*^) orbital and a lower energy bonding (*π*) orbital. The bonding electrons reside in the *π*‐orbital, the highest occupied molecular orbital (HOMO), leaving the *π* ^*^ orbital, the lowest unoccupied molecular orbital (LUMO) unoccupied. The addition of another pair of pz orbitals of the correct symmetry results in the formation of four new closely spaced molecular orbitals with a concomitant decrease in the HOMO‐LUMO gap. Such a trend is expected to continue with the sequential addition of monomer units, which ultimately results in the merging of the discrete energy levels, giving rise to a band structure that is reminiscent of that commonly observed in inorganic semiconductors (Figure 4C). There comes a point, termed the effective conjugation length (≈10–20 repeat units), beyond which, the bandgap becomes independent of further addition of repeat units (i.e., molecular weight, M_w_).^[^ ^41^ ^]^ The size of the bandgap, and hence the wavelength of light absorbed (and emitted), can be tuned to span the entire visible spectrum by controlling the extent of delocalization (i.e., the strength of bonding) and through the chemical design of the constituent monomers. For example, an electron‐rich monomer (donor) and an electron‐deficient monomer (acceptor) can be copolymerized to generate a donor‐acceptor (D‐A) polymer with a reduced bandgap. Here, the acceptor monomer will typically have a lower‐lying HOMO and LUMO when compared to the donor. Mixing of the orbitals with the correct symmetry of the respective monomers will subsequently generate hybrid molecular orbitals whereby the resulting HOMO is raised in energy and the LUMO is lowered in energy (i.e., an overall reduction in the bandgap).

Despite displaying superior optical properties, CPs are inherently hydrophobic, and this precludes their direct use as fluorescent probes for biological imaging and detecting biomolecules. One approach to combat this is to make CPs more hydrophilic by covalently linking hydrophilic side groups (e.g., PEO, PEG) or by functionalizing the side chains with ionic pedant groups.

SPNs exhibit exceptional per particle brightness, ≈100 times greater than that of similarly sized quantum dots (QDs), as a result of their large molar extinction coefficients (≈10^8^ M^−1^ cm^−1^), high chromophore density (≈10^5^) and moderate quantum yields (≈60% for visible emitting polymers and ≈3% for IR emitters).^[^ ^3^, ^42^, ^43^ ^]^ Consequently, concentrations of SPNs as low as 155 pM can be detected in cells, which bodes well for systems in which minimal cell perturbation is required.^[^ ^44^ ^]^ Further, SPNs have also been reported to exhibit excellent photostability with photon numbers exceeding 10^9^ before irreversible photobleaching is observed. This number far exceeds that of organic dyes and compares favorably with QDs.^[^ ^9^ ^]^ SPNs also exhibit fast radiative rates and minimal blinking behavior making them excellent probes for use in flow cytometry and single particle tracking.^[^ ^7^ ^]^ Lastly, SPNs have also been found to exhibit size‐independent optical properties, which makes them suitable (unlike QDs) for a wide range of biological applications including cellular labeling studies, sensing, and for in vivo imaging.^[^ ^9^ ^]^

To exploit the exceptional luminescent properties of SPNs in biological systems effectively, it is important to have control over their surface chemistry to render them biocompatible.^[^ ^5^ ^]^ Fortunately, the relatively benign nature of SPNs ensures that these fluorescent probes exhibit very little or no cytotoxicity, making them excellent fluorescent probes for cell labeling and imaging. For example, Christensen and co‐workers have shown that SPNs derived from PFBT (poly(fluorene‐alt‐benzothiadiazole), average diameter 18 ± 5 nm) were taken up by J774A.1 cells via an endocytic uptake mechanism and exhibited no significant cytotoxic or inflammatory effects.^[^ ^44^ ^]^

One particular optical attribute that differentiates SPNs from their inorganic counterparts (quantum dots) is their size‐independent optical properties. The emission wavelength and hence color are dictated by the constituent polymer rather than any size‐confined excitons: the only notable phenomenon is the increase in emission brightness with increasing particle size.^[^ ^45^ ^]^ This does not mean that particle size does not play a role in the emission wavelengths, although this is not attributed to the classic quantum size effects. No standard excitonic diameter has been calculated for these structures as their polymeric nature makes such descriptions and measurements unhelpful, although the exciton diffusion length has been measured in MEH‐PPV SPNs^[^ ^46^ ^]^ to be ≈12, and ≈19 nm in PFBT SPNs^[^ ^47^ ^]^ In several SPN systems, the tuning of the emission color has been achieved, although this is usually through carefully controlled synthesis using differing amount of reagents, hence inducing steric effects as a function of particle size.^[^ ^48^ ^]^ Tuning the emission across the visible region is possible, notably in MEH‐PPV, by controlled oxidation or the acid etching of the polymer leading to chain cleavage.^[^ ^49^, ^50^ ^]^

### 2.3 Application of SPNs

SPNs have a very large surface area, allowing targeting units to be attached to ensure that a sufficient number of SPNs are taken up by the cell and to minimize any non‐specific uptake, thereby reducing off‐site toxicity and false positives.^[^ ^5^ ^]^ In addition to targeting moieties, SPNs can also be conjugated with a variety of biologically relevant ligands that endow SPNs with the ability to cross different biological membranes (i.e., cell/tumor penetrating peptides).^[^ ^51^ ^]^ Targeting units can be introduced to SPNs by covalently attaching functional units to the backbone of the parent CP. For example, Chiu and co‐workers have prepared a series of CPs functionalized with side‐chain carboxylic groups to prepare SPNs and successfully bioconjugated streptavidin to the CPs for specific cellular labeling. Interestingly, the authors show that as little as 2.3% molar fraction of carboxylic acid surface units are required to perform bioconjugation and cellular labeling.^[^ ^52^ ^]^ A different, more versatile, and convenient approach is to noncovalently introduce the functional units into the polymer matrix in a similar way to that used for inorganic nanoparticles. For example, Howes and co‐workers have encapsulated SPNs with phospholipid micelles via a miniemulsion technique to generate colloidally stable SPNs that were further bioconjugated to bovine serum albumin to perform targeted imaging. In that study, the CP and the amphiphilic PEG‐lipid were co‐dissolved in a water‐immiscible solvent and subsequently transferred to an excess volume of water, resulting in micellar encapsulation.^[^ ^53^ ^]^

More recently, SPNs have been encapsulated with polystyrene maleic anhydride (PSMA) to endow the nanoparticles with surface active carboxylic acid units, which can take part in further reactions. For example, Wu and co‐workers have prepared surface functionalized SPNs for use in the biorthogonal labeling of intracellular proteins. The same researchers have also demonstrated the versatility of this approach by preparing functionalized SPNs for the targeting of cell surface markers and antibodies.^[^ ^8^ ^]^ The authors propose a mechanism by which the hydrophobic unit of the amphiphilic polymer embeds itself in the CP matrix and the anhydride units hydrolyze to the corresponding carboxylates. This process is mediated by hydrophobic interactions between the encapsulating polymer and the conjugated polymer and is therefore independent of the nature of the SPN.^[^ ^8^ ^]^ Other less common approaches for the noncovalent functionalization of SPNs include encapsulating the SPNs with a silica layer or by exploiting biological membranes to generate a biomimetic monolayer coating.^[^ ^51^, ^54^ ^]^

While outside the scope of the present review, SPNs have also been used in photothermal therapy (PTT), which leverages the ability of SPNs to convert light into thermal energy with very high photothermal conversion efficiency (PCE).^[^ ^55^ ^]^ For instance, Pan et al. utilized a near‐infrared (NIR) absorbing semiconductor polymer for both NIR fluorescence imaging and photothermal conversion. The design incorporated a gadolinium‐grafted triblock amphiphilic copolymer (F127‐DTPA‐Gd) to enable effective MR/fluorescence dual‐modal imaging. This research introduced a simple nanotheranostic platform for MR/fluorescence dual‐modal imaging‐guided PTT, which was used to inhibit the growth of oral squamous cell carcinoma in a mouse model.^[^ ^56^ ^]^ In addition to generating heat, SPNs can also undergo thermal expansion when irradiated with light to produce acoustic waves, which can be detected by an ultrasound transducer to produce an image, a technique known as photoacoustic imaging (PAI).^[^ ^2^, ^57^ ^]^ SPNs can also be employed as photosensitizers in photodynamic therapy (PDT), in which a light source is used to generate cytotoxic singlet oxygen and other reactive species to induce a therapeutic response.^[^ ^58^ ^]^ SPNs can also transfer energy (i.e., Förster resonance energy transfer, FRET) to photosensitizers (acceptors) such as phosphorescent iridium(III) complexes to amplify the PDT effect.^[^ ^59^ ^]^

### 2.4 Optical Imaging with SPNs

One of the most promising applications of SPNs is their use as fluorescent probes for molecular imaging to allow the non‐invasive visualization of biological processes in living systems. However, careful consideration is needed when designing SPNs for in vivo use as interference from tissue autofluorescence and light scattering severely affect the tissue penetration depth. To address this, there have been significant developments in the design of fluorescent probes to operate in the near‐infrared region (NIR), as tissues are typically transparent at these wavelengths (650–1350 nm), and consequently, there is minimal scattering and absorption of NIR light by biological tissue.^[^ ^60^ ^]^ Attempts to tune the emission profile of SPNs to the NIR usually involve modulating the emission spectra of SPNs through a rational donor‐acceptor design of the CP,^[^ ^61^ ^]^ doping with an NIR emitting dye^[^ ^13^, ^62^ ^]^ or by employing time‐resolved luminescence imaging that exploits the very long fluorescent lifetimes of NIR‐emitting SPNs.^[^ ^60^ ^]^ For example, SPNs have been doped with a NIR‐absorbing ^1^O_2_ sensitizer for afterglow luminescence and were found to exhibit an emission peak at 780 nm.^[^ ^13^ ^]^ Many more elaborate NIR probes have been reported that either modify the CPs directly or utilize FRET as a way of redshifting the emission spectrum.^[^ ^63^ ^]^ A more recent approach to overcoming limited tissue penetration in optical imaging was developed by Tan and coworkers.^[^ ^64^ ^]^ They use ultrasound to activate luminescent molecules through a two‐step energy conversion, in which ultrasound converts mechanical energy into chemical energy, generating strong luminescence with tissue penetration up to 2.2 cm, much deeper than conventional methods. While this technique shows significant potential, the detailed mechanisms of ultrasound‐induced luminescence and the molecular design principles for optimizing this effect are still not fully understood. Though showing promise, ultrasound‐triggered luminescence in biomedical imaging is still at an early stage of research.

Most design strategies to optimize optical imaging focus on enhancing the properties of the constituent conjugated polymers as, once the nanoparticle has been formed, it is very difficult to further optimize the optical performance. SPNs, in their current form, remain some of the most effective optical imaging agents available, due to their high brightness, stability, and available color palette, noticeably in the near‐infrared (NIR) spectral region. However, a reduction in the emission intensity is often seen when the particles are formed due to the change in polymer conformation and the formation of aggregates.^[^ ^38^ ^]^ Improvement in the optical properties can be realized by the inclusion of an antifade reagent,^[^ ^65^ ^]^ or the use of novel structures such as liquid cores that do not restrict the emitting polymer.^[^ ^66^ ^]^

As a result of these advances, semiconducting polymer nanoparticles (SPNs) are now widely recognized as a superior class of fluorophores, which exhibit excellent biocompatibility, extremely high brightness, and a tuneable emission profile.^[^ ^67^ ^]^ In addition, SPNs possess a rich surface chemistry through which ligands can be incorporated to further increase the functionality of SPNs. In recent years, there have been attempts to develop SPNs that operate in the NIR window but, despite improvements in the synthesis of NIR‐I/II probes, the moderate increase in penetration depth has hindered their use in a clinical setting. An alternative approach to address the issues surrounding tissue attenuation is to incorporate an additional non‐radiative imaging modality to complement the strengths of optical imaging.^[^ ^14^ ^]^ MRI offers many attributes that complement fluorescence imaging due to its non‐ionizing nature and its ability to yield images unrestricted by penetration depth.^[^ ^68^ ^]^

## 3 Magnetic Resonance Imaging

Magnetic resonance imaging (MRI) is a non‐invasive imaging modality that uses a powerful external magnetic field to obtain detailed anatomical and functional images with excellent soft tissue contrast and penetration depth. Through the influence of a strong external magnetic field, the magnetic moment of hydrogen nuclei will align parallel to that of the external magnetic field. The application of a radiofrequency excitation event then tips the magnetization onto the XY plane; producing a detectable signal from which qualitative images may be generated. Upon the removal of this excitation event, the hydrogen nuclei undergo a process called relaxation, which occurs via two independent processes: *T_1_* relaxation (spin‐lattice or longitudinal) and *T_2_* (spin‐spin or transverse). The time taken for each of these processes to occur is referred to as the *T_1_* or *T_2_* relaxation time, respectively.^[^ ^69^ ^]^ During an MRI scan, hydrogen nuclei residing in different tissues will have distinct relaxation times and, by exploiting the variation in concentration and relaxation times of the spin active nuclei, an image can be produced.

Compared to other imaging modalities (e.g., PET, optical), MRI suffers from poor sensitivity, and attempts to improve this aspect typically involve the use of high‐field MRI scanners (e.g., 7T) to increase the population of protons that are aligned with the magnetic field. Another related issue is the poor endogenous contrast between different soft tissue types, which has led to the widespread administration of a contrast agent (CA) to accelerate the relaxation rates of neighboring water protons to improve image contrast.^[^ ^70^ ^]^ Contrast agents can be divided into two categories depending on their mode of mechanism, namely *T_1_* CAs and *T_2_* CAs. The efficacy of *T_1_* and *T_2_* CAs to accelerate the relaxation rate of neighboring water protons can be expressed by the relaxivity terms *r_1_* and *r_2_*, respectively. The relationship between relaxivity and the relaxation rate is shown in Equation 1 where *T_i,obs_* refers to the observed relaxation time of the hydrogen nuclei, *T_i,d_* is the relaxation time in the absence of the contrast agent and [CA] refers to the concentration of the contrast agent.

(1) $$\frac{1}{T_{i,obs}}=\frac{1}{T_{1,d}}+r_{i}\left[\mathrm{CA}\right]\left(i=1,2\right)$$

Although a contrast agent (CA) can shorten both *T_1_* and *T_2_*, the ratio between *r_1_* and *r_2_* is an important parameter to consider when determining the preferred mode of relaxation. Conventionally, if the ratio *r_2_*/*r_1_* is less than 2, the contrast agent is considered a *T_1_* CA. If this ratio is greater than 10 then the contrast agent is considered to be a *T_2_* agent, whilst those in between are considered to be dual modal. An important consideration is that *T_1_*‐weighted contrast agents (such as those based on gadolinium) lead to a brighter image where the agents are localized, while *T_2_*‐weighted contrast agents (such as SPIOs) result in a darkening of the area in which the CAs are present. The former is generally preferred in a clinical setting.

### 3.1 Gadolinium‐Based Contrast Agents

The most commonly used *T_1_* contrast agents for MRI are those based on the highly paramagnetic gadolinium ion, Gd^3+^, which exhibits long electron‐spin relaxation times as a result of the symmetry of the Gd^3+^ ground state (S = 7/2). However, despite being one of the most potent ions for generating *T_1_* relaxation, the inherent insensitivity of MRI requires a bolus injection of this contrast agent. The alarmingly large doses of Gd^3+^ required to deliver the required contrast enhancement and the innate toxicity of free Gd^3+^ ions have raised concerns over the safety of gadolinium‐based contrast agents (GBCAs). Unbound Gd^3+^ ions have been linked to an increased risk of nephrogenic systemic fibrosis (NSF) and may also interfere with calcium‐dependent pathways. As a result, some limitations have been placed on the use of GBCAs preventing them from being used in patients suffering from severe renal insufficiency and acute kidney injury.^[^ ^68^ ^]^ Despite the concerns, ≈40% of clinical MRI scans employ CAs based on Gd^3+^ chelates owing to the dramatic improvement in diagnostic performance,^[^ ^71^ ^]^ and the resulting benefits for patient outcomes. The toxicity of GBCAs is minimized by binding the Gd^3+^ ions to cyclic chelators based on the thermodynamically and kinetically stable polyaminocarboxylate motif. An additional and very promising strategy is to combine this encapsulation with improvements in the relaxivity performance per Gd^3+^ ion in order to reduce the dose needed. The polymer‐based materials discussed in this review exploit the limited rotational freedom of the attached Gd units that results in dramatic enhancement of their performance.

**Figure 5:**
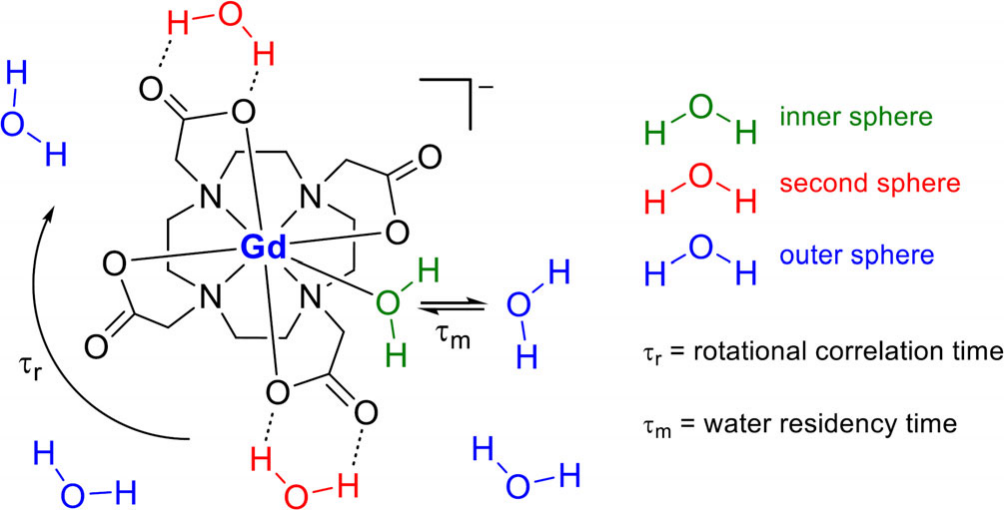
Overview of the molecular parameters contributing to the relaxivity, *r_1_*, based on Dotarem.

*T_1_* relaxation occurs via a dipolar mechanism whereby the fluctuating magnetic dipole of a paramagnetic center induces a spin relaxation of the hydrogen nuclei. The coupling interaction between the paramagnetic ion and the hydrogen nuclei is distance‐dependent. As a result, the interaction with the water molecules can be categorized using three categories: i) inner sphere, where the water molecule is directly bound to the metal center; ii) second sphere, where the water molecules diffuse around the metal center with a finite residency time that is longer than the translational diffusion of pure water and iii) outer sphere, where water molecules freely diffuse around the metal center and can be characterized by the translational diffusion time (**Figure** **5**).^[^ ^72^ ^]^

The contribution from the inner sphere water molecules to relaxivity is dominant for most small‐molecule contrast agents and three molecular parameters can be identified, which can be modified to improve the inner sphere relaxivity of the paramagnetic metal. These are the hydration number (*q*), the water residence lifetime (τ_m_), and the rotational correlation time (τ_r_). All three can be enhanced through careful ligand design, while the last of these can be influenced by attaching the metal to a larger structure to slow down the rotational motion. In most cases, an increase in the hydration number correlates with a decrease in thermodynamic and/or kinetic stability with respect to transmetallation (displacement of the Gd^3+^ ion by endogenous metals) and, as a result, most reported gadolinium chelates are based on monohydrated complexes (*q* = 1). Stable gadolinium chelates with larger hydration numbers (*q* = 2 or 3) have been reported but these complexes often exhibit reduced relaxivity values due to the displacement of the water molecules by endogenous ligands such as carbonate, bicarbonate, and phosphate.^[^ ^73^ ^]^

Attempts to modify both t_m_ and *r_M_*_‐H_ usually involve complex chemical derivatisations and, in most cases, the t_m_ of most exogenous contrast agents are already within their optimal range. Further, the majority of Gd complexes have been reported to have very similar *r_Gd‐H_* distances of 3.1 Å from ^1^H ENDOR analysis.^[^ ^72^ ^]^ In contrast, the t_r_ values for most Gd^3+^‐based CAs lie in a sub‐optimal range, which offers an avenue for the improvement of their relaxivity.

### 3.2 Gd‐Functionalised SPNs as *T_1_* Contrast Agents

**Figure 6:**
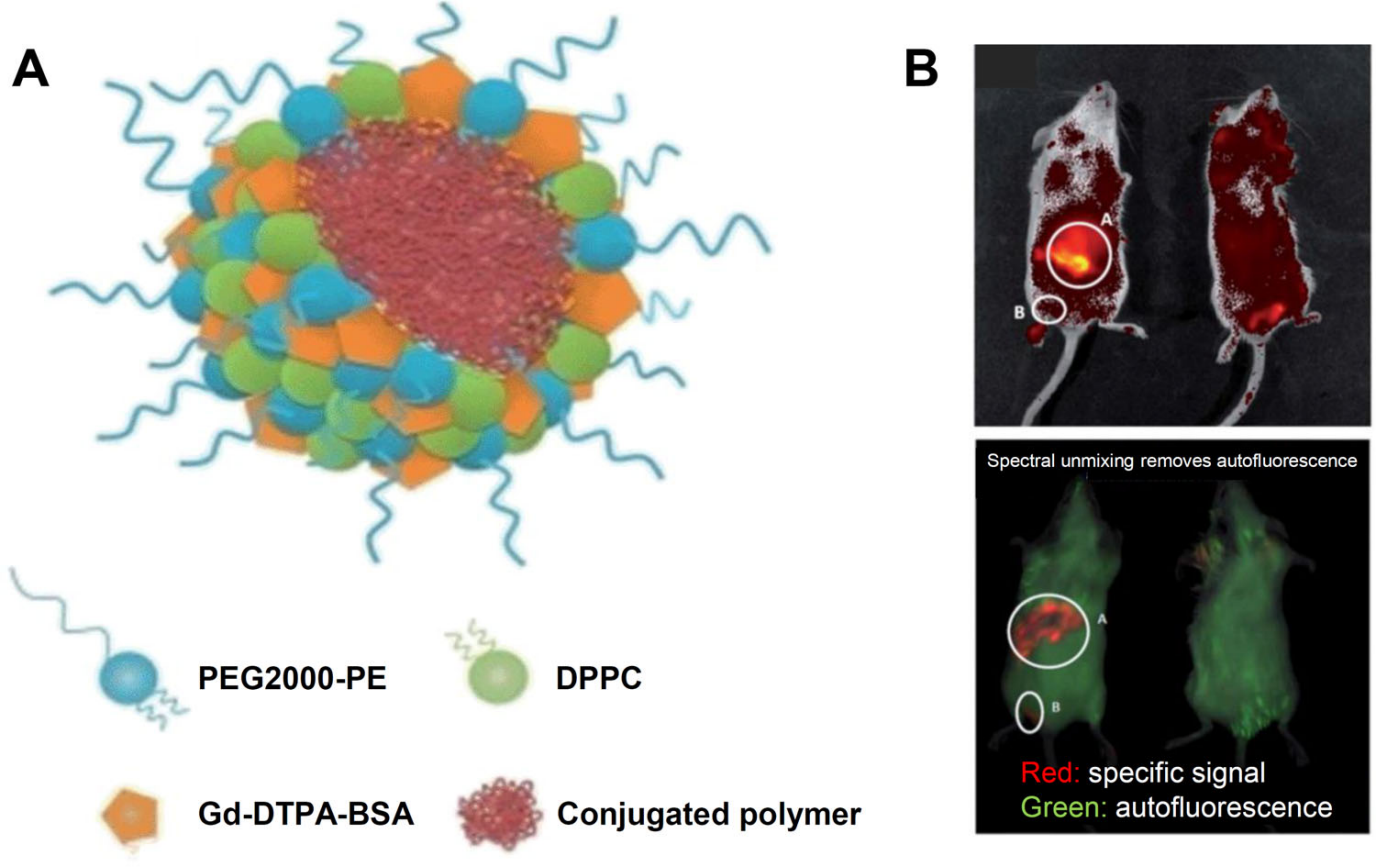
A) Bimodal SPNs prepared with Gd‐DTPA‐BSA combined with the CPs, PPE, MEH‐PPV, F8BT or ADS108GE and B) images of mice injected with A) 100 mL MEH‐PPV Gd‐SPNs subcutaneously on the ventral surface and B) 100 mL MEH‐PPV (Gd‐SPNs)‐IgG intramuscular into the quadricep muscle; the upper image is an IVIS image showing the collected fluorescence intensity image against the mice ambient image and the lower image is an IVIS processed image that shows the fluorescence from the nanoparticles (red) and the animal's autofluorescence (green). Reproduced (adapted) with permission.^[^ ^74^ ^]^ Copyright 2014, Royal Society of Chemistry.

The τ_r_ value for gadolinium(III) CAs can be increased by attaching the lanthanide to the surface of globular structures such as SPNs in order to slow down their tumbling rates. For example, Gd^3+^ units were successfully attached to the surface of SPNs derived from poly[2‐methoxy‐5‐(2‐ethylhexyloxy)‐1,4‐phenylenevinylene] (MEH‐PPV) by covalently linking a Gd‐diethylenetriaminepentaacetic acid bis(stearylamide) gadolinium complex (Gd‐DTPA‐dBSA) to a phospholipid, which was then used to encapsulate the SPNs via a miniemulsion method. The resulting SPNs (hydrodynamic radius (*D_h_*) between 111–117 nm) had an absorption maximum at ≈495 nm and an emission peak centered at 592 nm. The SPNs were found to be readily taken up by both HeLa and J774 cells, and preliminary in vivo studies in mice demonstrate the utility of these probes for optical imaging (**Figure** **6**). Further, the SPNs were found to exhibit an *r_1_* value of 20.8 mM^−1^ s ^−1^ per Gd^3+^ ion at the clinical field strength of 3 T (and 17.2 mM^−1^ s ^−1^ at 7 T), highlighting the potential of these probes for dual‐mode imaging. Impressively, this *r_1_* value represents a 6‐fold increase when compared to the clinical “gold standard,” Dotarem. Subcutaneous injection of MEH‐PPV Gd‐SPNs in mice resulted in strong fluorescence visible through the skin. Intramuscular injection of antibody‐conjugated MEH‐PPV Gd‐SPNs at 1000x lower concentrations still produced detectable fluorescence. However, it was found that deep chest cavity injections yielded no detectable signal due to tissue attenuation, which further confirmed that the fluorescence intensity decreases with injection depth and depends on nanoparticle concentration and targeting conjugation.^[^ ^74^ ^]^

**Figure 7:**
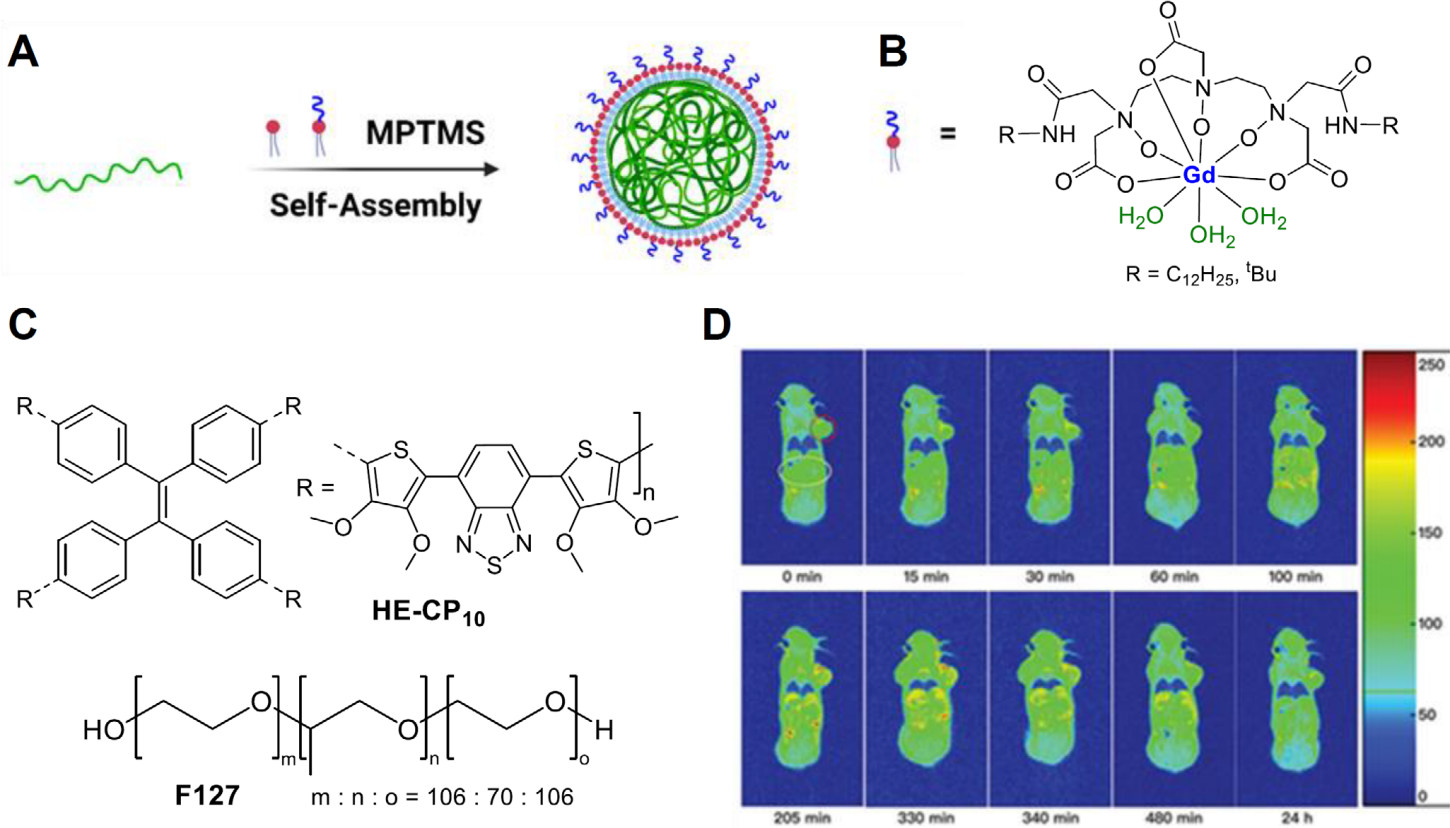
A) Self‐assembly of lipid vesicles containing Gd‐DTPA‐NO‐(C_12_H_25_)_2_, F127 (surfactant) and the CP, HE‐CP_10_, with crosslinking was performed with (3‐mercaptopropyl)trimethoxysilane (MPTMS); B) Metallosurfactant, Gd‐DTPA‐NO‐(C_12_H_25_)_2_; C) structures of HE‐CP_10_ and F127; D) *T_1_* weighted images of nude mice taken at different time points after injection of SPNs. Circles indicate regions of the tumor (red) and kidney (yellow). Reproduced (adapted) with permission.^[^ ^75^ ^]^ Copyright 2020, WILEY‐VCH Verlag GmbH & Co. KGaA, Weinheim.

To further improve the relaxivity performance and to produce smaller nanoparticles, a novel red‐emitting CP was used to prepare SPNs which were then incorporated into a mixed phospholipid system containing Gd‐DTPA‐NO‐(C_12_H_25_)_2_ units (*q* = 3) via a matrix encapsulation method (*cf*. nanoprecipitation). The mixed surfactants and conjugated polymers were dissolved in tetrahydrofuran and rapidly injected into an excess of water to yield lipid vesicles of ≈94 nm in diameter (**Figure** **7**A). Here, a strategy to improve the relaxivity by simultaneously increasing the hydration number of the Gd^3+^ unit (*q* = 3) and slowing down the tumbling rate through the self‐assembly of the surfactant units into the NP structure was employed.^[^ ^75^ ^]^ However, metallosurfactants such as Gd‐DTPA‐NO‐(C_12_H_25_)_2_ (Figure 7B; R = C_12_H_25_) are prone to rotational diffusion on the surface of lipid vesicles, which increases the overall rotational correlation time of the Gd^3+^ unit, resulting in a reduced relaxivity gain.^[^ ^76^ ^]^ To circumvent this, the mixed phospholipids were cross‐linked providing a more rigid system and this led to the extremely large reported *r_1_* value of 105.4 mM^−1^ s^−1^ per Gd ion at 1.5 T (Figure 7C).^[^ ^75^ ^]^ As a control, SPNs encapsulated with Gd‐DTPA‐(C_12_H_25_)_2_ (*q* = 1) were also prepared, yielding an *r_1_* value of 40.8 mM^−1^ s^−1^ per Gd unit at 1.5 T, which suggests that the main contributing factor to the enhancement of the relaxivity was the increase in hydration number (Figure 7B). To demonstrate the potential of these probes as CAs for tumor‐targeted magnetic resonance imaging, nude mice bearing subcutaneous human breast tumors (MDA‐MB‐231) were intravenously injected with the probe, and *T_1_*‐weighted images were acquired. As expected, the SPNs were found to display prolonged tumor retention owing to the enhanced permeability and retention (EPR) effect and displayed good MRI signal enhancements even 24 h post‐injection (Figure 7D).^[^ ^75^ ^]^

Despite the increase in hydration number, the complexes were shown to exhibit similar formation constants (log*K_GdL_* = 19.35) when compared to other commercially available MRI contrast agents (log*K_GdL_* = 22.46).^[^ ^77^ ^]^ However, a more judicious comparison would involve an assessment of the kinetic inertness of the complexes as this has been shown to be a more useful predictor of in vivo Gd^3+^ release. As a proxy for assessing the kinetic stability, it has been shown that the parent complex Gd‐HAO‐2 (Figure 7B; R = ^t^Bu) exhibits kinetic inertness comparable to that of other acyclic systems such as the recently suspended Omniscan. However, the kinetic stability is much lower than that of Gadolinium tetraazacyclododecanetetraacetic acid (Gd‐DOTA), which is considered the “gold standard” for contrast‐enhanced MRI imaging.^[^ ^77^ ^]^ This suggests that Gd‐DTPA‐NO‐(C_12_H_25_)_2_ may also exhibit similarly poor kinetic inertness, which could adversely impact its clinical translation. The withdrawal from clinical use of Omniscan due to toxicity concerns has led to a widespread reluctance to use gadolinium complexes with acyclic chelators and so this must be added as a design consideration for new Gd‐functionalised SPNs intended for use as contrast agents in MRI. This is further reinforced by studies that rely on the availability of residual carboxyl groups of the polymeric nanoparticles to chelate the Gd^3+^ ions in an undefined manner to provide contrast enhancement.^[^ ^78^ ^]^

Polypyrrole (PPy) is a low‐bandgap CP, which additionally provides the resultant SPN with the option of phototherapeutic action on excitation with near‐IR laser light. A typical example of a PPy‐derived SPN is that designed by Dai, Liang, and coworkers for dual PA‐MRI (but not fluorescence) imaging. An aqueous dispersion polymerization method was used to prepare the SPN by mixing PPy and pyrrole‐1‐propanoic acid (PPy‐COOH) in the presence of FeCl_3_ before conjugation to a DOTA derivative via a bis(amine)‐PEG linker (NH_2_‐PEG‐NH_2_).^[^ ^79^ ^]^ Subsequent Gd^3+^ coordination to the SPN yielded Gd‐PEG‐PPy, which displayed a relaxivity (*r_1_*) value of 10.6 mM^−1^s^−1^ per Gd unit at 3T, which is significantly lower than most other reported nanoparticle‐based systems.^[^ ^77^ ^]^ This illustrates that the use of longer linkers, such as polyethylene glycol (PEG), to attach the Gd unit to a larger assembly can undermine to some degree the potential significant relaxivity gain from immobilization. This can be traced to the fact that the Gd^3+^ unit retains much of its rotational freedom when attached via a long linker, reducing the relaxivity gain from conjugation to the large particle. In addition, the crowding effect of the PEG chains can also prevent water access to the metal center, reducing the ability of the coordinated water molecules to exchange rapidly with the bulk water. The strategy of first creating the nanoparticle and then subsequently adding gadolinium ions to the empty chelates may also result in Gd^3+^ ions remaining loosely bound to the structure if only limited purification steps are employed. It is imperative that the toxicity of free gadolinium ions is considered at the design stage and that rigorous purification steps are used to remove any unbound Gd ions.

**Figure 8:**
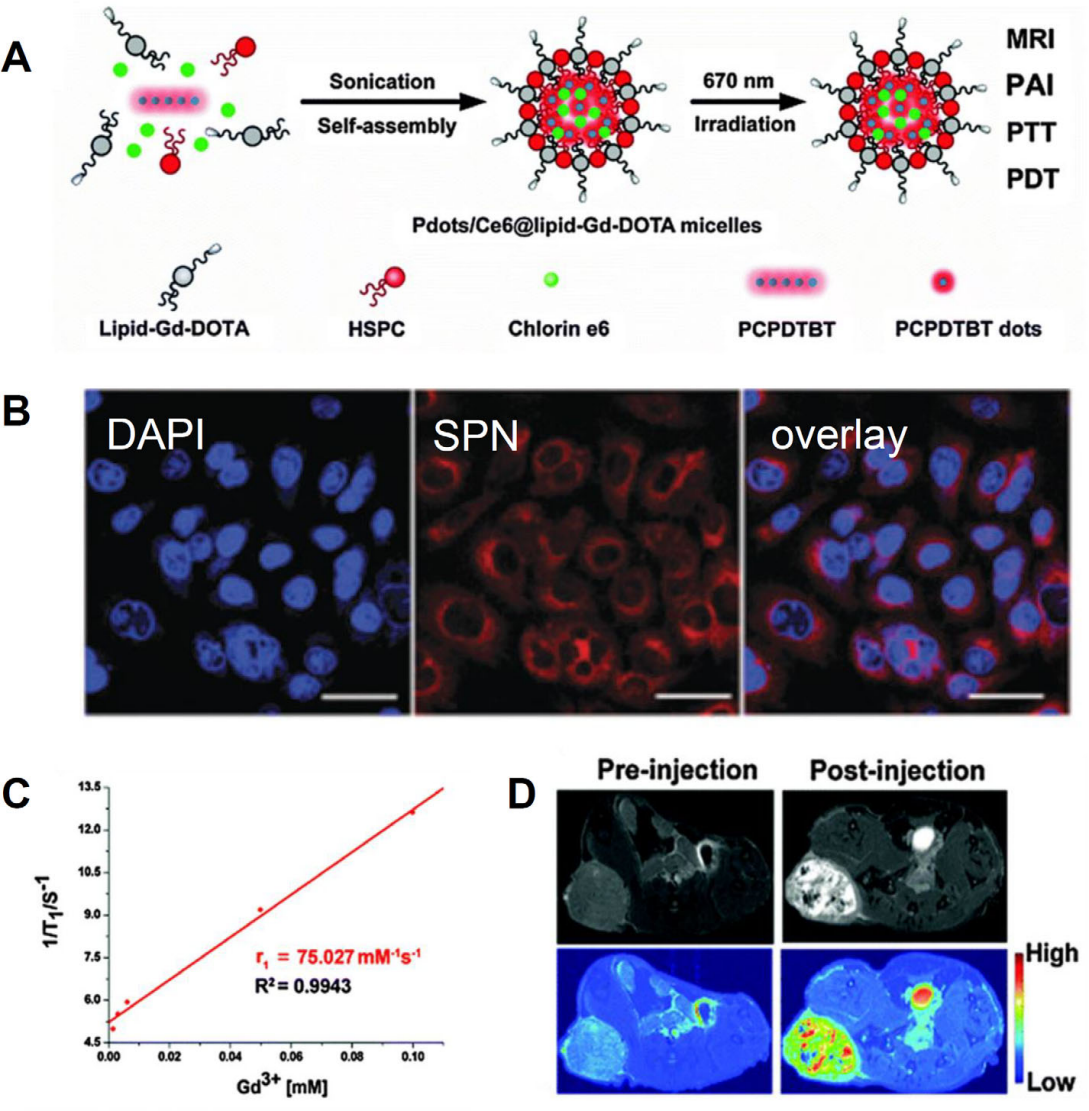
A) Self‐assembly of SPNs with Gd‐DOTA‐PEG‐DSPE (polyethylene glycol‐1,2‐distearoyl‐sn‐glycero‐3‐phosphorylethanolamine), HSPC (1‐palmitoyl‐2‐stearoyl‐sn‐glycero‐3‐phosphatidylcholine), PCPDTB (poly[2,6‐(4,4‐bis‐(2‐ethylhexyl)‐4H‐cyclopenta[2,1‐b;3,4‐b′]‐dithiophene)‐4,7‐(2,1,3‐benzothiadiazole); B) Confocal images of HepG2 cells incubated with the SPNs for 3 h; C) a plot of *R_1_* (1/*T_1_*) as a function of the Gd^3+^ concentration in SPN/Ce6@lipid–Gd–DOTA; D) *T_1_* weighted images of nude tumor‐bearing mice before and after injection with SPN/Ce6@lipid–Gd–DOTA.^[^ ^80^ ^]^ Reproduced (adapted) with permission.^[^ ^80^ ^]^ Copyright 2016, Royal Society of Chemistry.

A mixed phospholipid system containing Gd‐DOTA‐PEG‐DSPE (DSPE = polyethylene glycol‐1,2‐distearoyl‐sn‐glycero‐3‐phosphorylethanolamine) was prepared to encapsulate the NIR absorbing polymer (poly(cyclopentadithiophene‐alt‐benzothiadiazole), PCPDTBT) that is commonly used to fabricate SPNs for use in photothermal therapy (PTT). Following the incorporation of chlorin e6, a photosensitizer commonly used in photodynamic therapy (PDT), into the core matrix of the SPN, a multifunctional nanoparticle SPN/Ce6@lipid–Gd–DOTA (**Figure** **8**A) was prepared that combines both PTT and PDT into one platform. Enhanced tumor‐killing efficiency was observed for this nanomaterial when combining both photodynamic therapy (PDT) and photothermal therapy (PTT) into a system requiring irradiation with only a single 670 nm laser. The resulting nanoparticles were also found to have a *D_h_* of 111.5 ± 3.16 nm and showed clear fluorescence in confocal images of HepG2 cells incubated with the SPNs (Figure 8B). Just as importantly, the multifunctional nanoparticle was reported to achieve an unusually high *r_1_* value of 75.0 mM^−1^s^−1^ at 9.4 T, which warrants further investigation (Figure 8C).^[^ ^80^ ^]^ Indeed, the theoretically maximum *r_1_* value at 9.4 T for monohydrated gadolinium(III) complexes with optimized parameters is reported to be just 18 mM^−1^ s^−1^.^[^ ^81^ ^]^ As expected, *T_1_*‐weighted MRI images of nude mice bearing the HepG2 tumor were found to exhibit significant brightening following the injection of the multifunctional nanoparticles (Figure 8D).

There have also been examples reported in the literature for monohydrated gadolinium complexes which appear to exhibit relaxivity values close to, or even in some cases exceeding the theoretical limit. In such cases, a significant second sphere contribution was invoked to account for the enhanced relaxivity values. For example, it was recently shown that monohydrated gadolinium(III)‐DNA conjugates deposited in the curvatures of gold nanostars exhibit *r_1_* values of up to 98 mM^−1^s^−1^ at 32 MHz. Based on nuclear magnetic relaxation dispersion (NMRD) analysis, the authors suggest that the gadolinium(III) contrast agent that bears extended hydrophilic polymer or protein surfaces, provides a hydrogen‐bonding‐rich environment, which slows down the diffusion of water molecules and consequently increases the second sphere contribution to relaxation.^[^ ^82^ ^]^ In some cases, the inner sphere relaxation contribution was shown to be negligible when a large contribution arises from the second sphere. For example, hydrogels functionalized with a gadolinium complex containing no inner sphere water molecules [Gd(DOTP)]^5−^ were reported to exhibit surprisingly large *r_1_* values (100 mM^−1^ s^−1^ at 30 MHz).^[^ ^83^ ^]^ It was proposed that the high values could be explained by a network of hydrogen‐bonding interactions resulting in a significant second sphere contribution to relaxivity. A similar explanation was used to explain the high relaxivity values reported for nanogels formed from chitosan and hyaluronic acid that incorporated [Gd(DOTP)]^5−^ units. The remarkable performance was attributed to the presence of strong and long‐lived hydrogen bonding interactions between the metal complex and second‐sphere water molecules.^[^ ^84^ ^]^ However, to date, no examples have been published that have sought to increase the second sphere contribution to relaxivity in the case of SPNs.

### 3.3 Gadolinium‐Free MRI Contrast Agents

**Figure 9:**
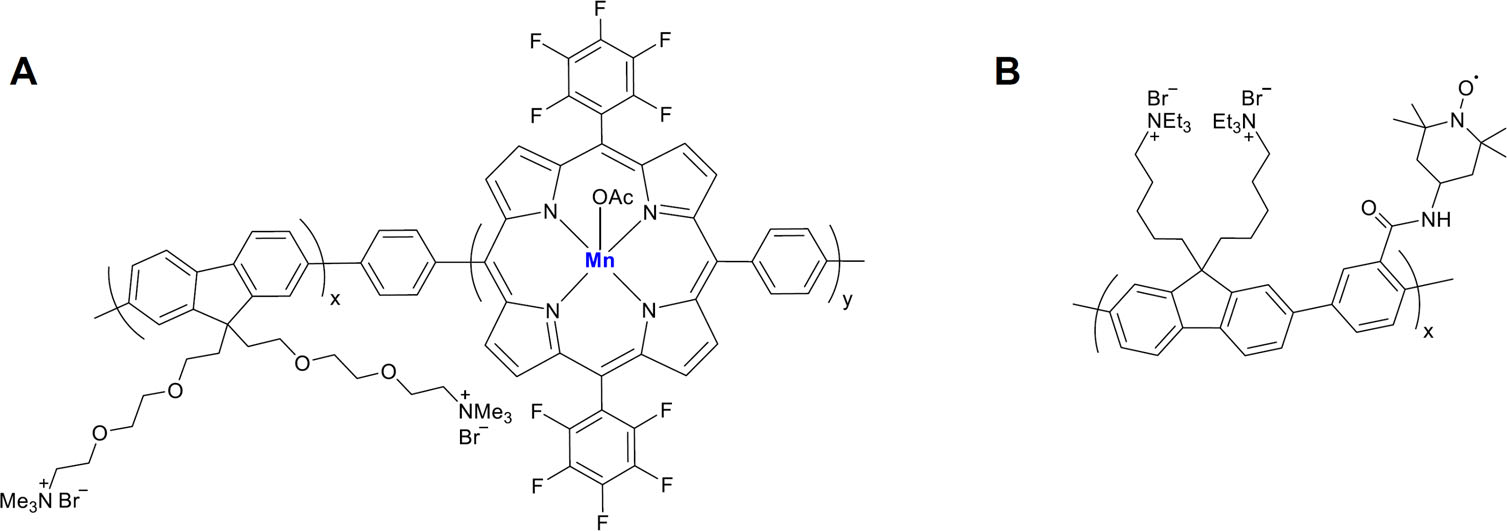
Examples of gadolinium‐free MRI contrast agents prepared from the self‐assembly of A) Mn(III) porphyrin‐incorporated polymer electrolyte^[^ ^88^ ^]^ and B) Conjugated polymer containing an organic radical.^[^ ^91^ ^]^

There have been recent attempts to investigate alternatives to gadolinium‐based CAs and to explore other metals for use as *T_1_* contrast agents. Most of this work has been focused on divalent manganese systems due to the 5 unpaired electrons that are symmetrically distributed in the five d‐orbitals of the metal,^[^ ^85^ ^]^ while the natural occurrence of manganese in the body allows the body to process and remove an excess of the metal.^[^ ^86^ ^]^ However, due to the challenge of stabilizing the divalent oxidation state of manganese,^[^ ^87^ ^]^ research has also been directed at trivalent Mn‐based contrast agents with 4 unpaired electrons. One such example is that of an SPN decorated with manganese porphyrin units through the self‐assembly of a CP‐based amphiphilic polymer (**Figure** **9**A). The absorption spectra for the resulting SPNs exhibit a dominant absorption band centered at 377 nm along with two minor peaks originating from the manganese(III) porphyrin units. On excitation at 375 nm, the SPNs emitted an intense blue fluorescence with an emission peak centered at 475 nm. Further, the SPNs were found to be non‐toxic and readily taken up by HeLa cells. Confocal microscopy demonstrated the potential of these probes for cell imaging as they were found to be exceptionally bright owing to their high quantum yield of 31%. However, the resulting SPNs only exhibited a relaxivity (*r*_1_) value of 13.2 mM^−1^ s^−1^ per metal unit at 0.55 T, which is significantly lower than other comparable nanoparticle‐based systems.^[^ ^88^ ^]^ The low *r_1_* value can be attributed to Mn^3+^ having only 4 unpaired electrons, which are not symmetrically distributed across the d‐orbitals.

It is also likely that the self‐assembly of the porphyrin‐functionalized CP restricts water access to the metal center and thereby reduces the overall relaxivity.^[^ ^89^ ^]^ As a comparison, the attachment of a *meso*‐tetraphenylporphyrin Mn(II) complex to the surface of a doxorubicin‐loaded polylactic acid (PLA) nanoparticle through an amide bond resulted in a relaxivity value of 27.9 mM^−1^s^−1^ at 0.5 T.^[^ ^90^ ^]^

In an attempt to move away from metal‐containing contrast agents, SPNs based on a CP functionalized with 2,2,6,6‐tetramethylpiperidine‐1‐oxyl (TEMPO) units have been developed (Figure 9B).^[^ ^91^ ^]^ Nitroxide organic radical CAs (ORCAs), such as TEMPO are generally considered to exhibit minimal toxicity and have been extensively used as CAs for redox sensing owing to the relative ease with which they are reduced to diamagnetic hydroxylamines.^[^ ^91^, ^92^, ^93^, ^94^, ^95^ ^]^ Due to their favorable toxicity profile, there have been attempts to incorporate multiple nitroxyl groups per polymer/particle in order to improve the relaxivity performance. For example, TEMPO‐based SPNs incorporating multiple nitroxyl groups were found to exhibit only a low relaxivity value of 0.28 mM^−1^ s^−1^ at 3.0 T.^[^ ^91^ ^]^ This low relaxivity value can be attributed to the smaller number of unpaired electrons in the organic radical compared to gadolinium(III) (7 unpaired electrons) or Mn(II) (5 unpaired electrons) in metal‐based CAs. An additional complication is the fact that TEMPO is known to undergo a facile reduction to become diamagnetic and hence MRI inactive, which can also reduce the overall relaxivity observed. Indeed, this is the basis for the same approach being used to detect the superoxide anion in biological environments.^[^ ^96^ ^]^ The use of TEMPO and similar nitroxyl‐based ORCAs may also adversely affect the fluorescent properties of the SPN as these species are also known to be efficient fluorescence quenchers.^[^ ^95^ ^]^

### 3.4 *T_2_*‐Weighted Contrast Agents

Superparamagnetic iron oxide nanoparticles are a widely investigated class of *T_2_*–weighted MRI CAs that combine good sensitivity with minimal toxicity at concentrations much higher than that needed for imaging.^[^ ^97^ ^]^ Several SPIONs have been approved by the FDA and EMA for use as *T_2_*–weighted MRI CAs, including Endorem and Resovist,^[^ ^98^ ^]^

Although their use purely for MRI contrast has declined due to the fact that *T_2_*–weighted images appear darker (rather than lighter as is the case for *T_1_*–weighted CAs), they are straightforward and attractive platforms for multimodal applications. Moreover, SPIONs are known to be cleared rapidly by macrophages, which significantly hampers their clinical translation, though the inclusion of an additional imaging modality may serve to improve their pharmacokinetics and consequently their use as *T_2_*‐weighted probes for MRI imaging.^[^ ^99^ ^]^ When placed in an external magnetic field, SPIONs induce a local magnetic field inhomogeneity, which shortens the *T_2_* relaxation rate of neighboring protons.

An important aspect particularly when considering the incorporation into polymeric materials is the impact of the surface coating on the relaxivity, *r_2_*. A highly hydrated surface coating will slow down the diffusion of the local water molecules, which in turn increases the local water concentration, and thus the *r_2_*. A smaller surface coating thickness can also favor faster *T_2_* relaxation by minimizing the distance between the SPIONs and the water Additionally, the surface coating has also been shown to play an important role in determining the arrangement of surface atoms, which can influence their surface properties (magnetic dead layer, spin structure, valence state, surface phase, etc.). Coating the surface of the SPION can alter the proportion of magnetically inactive regions at the surface, resulting in either an increase or a loss of magnetization near the surface. This effect is known as spin canting and this is a parameter that can be exploited in the pursuit of high *T_1_* contrast agents. In addition, the overall shape and size of the nanomaterial also influence the effective magnetization as this leads to non‐negligible spin canting effects.^[^ ^100^ ^]^ For example, Wei and co‐workers have prepared very small (1 nm) iron oxide nanoparticles that exhibit an *r_1_* value comparable to that of Gd‐DTPA. Interestingly, the nanoparticles were able to retain their magnetic properties when coated with a zwitterionic ligand to impart colloidal stability across a range of biologically relevant conditions.^[^ ^101^ ^]^ A recent study by Song and coworkers employed a polymer coating to stabilize nanoparticles and regulate their aggregation state, which directly affects the T_2_ relaxation time in MRI.^[^ ^102^ ^]^ When nitric oxide (NO) cleaves the linker, the structure of the polymer coating changes, altering nanoparticle clustering and resulting in a significant shift in the T_2_ signal. This modulation of T_2_ by the polymer coating enables highly sensitive and selective detection of NO in vivo, facilitating imaging and quantification of NO in complex biological environments.

**Figure 10:**
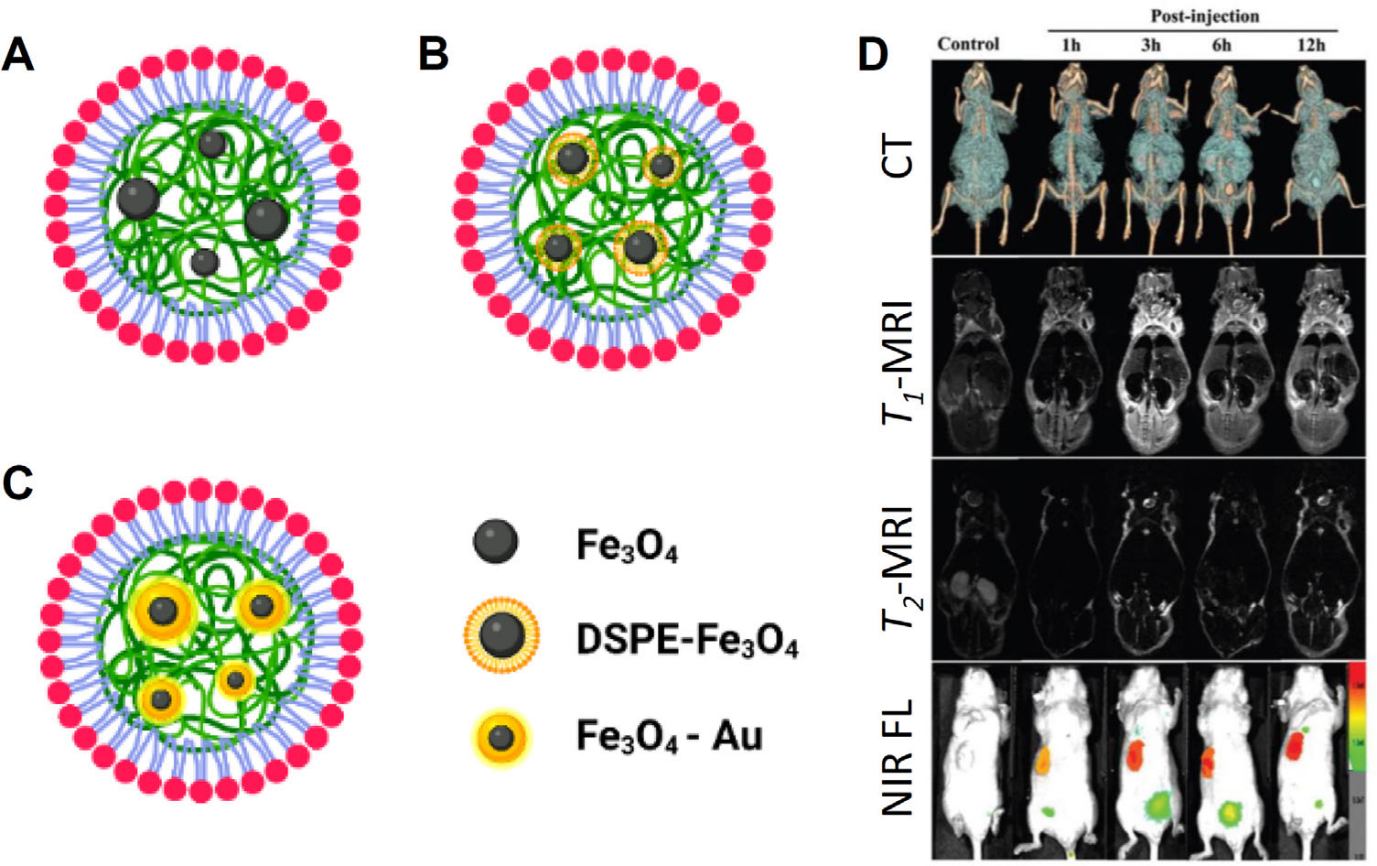
Examples of SPN CAs for *T_2_*‐weighted imaging. A) encapsulation of bare SPIONs;^[^ ^103^ ^]^ B) encapsulation of SPIONs coated by DSPE;^[^ ^105^ ^]^ C) encapsulation of SPIONs by a gold layer;^[^ ^106^ ^]^ D) In vivo tumor‐targeted CT/*T_1_* &*T_2_*‐MRI/NIR fluorescence trimodality imaging using the system shown in C of HepG2 tumor‐bearing mice before and after injection via the tail vein.^[^ ^106^ ^]^ Reproduced (adapted) with permission.^[^ ^106^ ^]^ Copyright 2018, Royal Society of Chemistry.

In an attempt to combine the magnetic properties of SPIONs with the favorable optical properties of SPNs, Howes and co‐workers developed hybrid magnetic‐fluorescent SPNs through the encapsulation of SPNs and SPIONs in phospholipid micelles (**Figure** **10**A).^[^ ^103^ ^]^ To do this, the CPs, the phospholipids, and the pre‐prepared SPIONs were all dissolved in dichloromethane (DCM) and transferred to water with continual stirring to induce droplet formation. Due to their amphiphilic nature, the phospholipids orientate themselves in such a manner that hydrophobic segments remain in the DCM phase and their hydrophilic segments protrude into the aqueous phase. Following evaporation of the DCM, the droplet shrinks in size to yield the magnetic nanomaterial. It was found that the incorporation of SPIONs into the core of the SPNs quenches the fluorescence emission of the SPN. Nonetheless, the authors were able to demonstrate a novel strategy for the incorporation of SPIONs into the core of the SPN and this led to an *r_2_* value of 152 mM^−1^ s^−1^ at 3.0 T. This unexpectedly large *r_2_* value may be traced to the *π*‐electron rich environment surrounding the SPIONs, which has previously been shown to increase *T_2_* relaxation by increasing the inhomogeneity of the local magnetic field.^[^ ^104^ ^]^ However, the wide variability in the amount of SPIONs encapsulated and the absence of measurement of effective magnetization value prevents a more robust comparison from being made.

To overcome the quenching of the fluorescence by the iron oxide, Li and co‐workers coated SPIONs with phospholipids (Figure 10B) to physically separate them from the SPNs and the authors then performed a series of in vivo fluorescence and magnetic studies.^[^ ^105^ ^]^ The authors demonstrated an approximate 2‐fold improvement in the fluorescence intensity by coating the SPIONs with 1,2‐distearoyl‐sn‐glycero‐3‐phosphorylethanolamine (DSPE). However, it was found that the SPNs displayed a relatively low r_2_ value of 55.2 mM^−1^ s^−1^ at 7.0 T. In the absence of a control lacking a lipid coating, the impact of the lipid coating on the magnetization cannot be determined unambiguously, although it is expected that the additional separation due to the lipid coating may adversely affect water accessibility. Further, the SPIONs in this study were prepared using a coprecipitation method, which typically yields SPIONs with low effective magnetization.

A more elaborate attempt to retain the fluorescence of SPIONs doped with SPNs was reported by Wang and co‐workers. In this work, ultra‐small SPIONs were coprecipitated with a gold shell to generate highly crystalline magnetic gold nanoparticles (Figure 10C).^[^ ^106^ ^]^ A solvent‐mediated method was subsequently used to reprecipitate the nanomaterials in the presence of a CP, a NIR‐emitting dye, and polystyrene maleic anhydride, to generate a trimodal system. The authors proposed a strategy in which the SPNs can simultaneously generate contrast‐enhanced images in MRI (*T_1_* and *T_2_*), fluorescence, and CT imaging (Figure 10D). This trimodal system was reported to exhibit r_1_ and r_2_ values of 6.64 and 27.93 mM^−1^s^−1^ at 7T, respectively. The low *r_2_* value was attributed to the small size of the SPIONs, which exhibit a greater degree of spin canting and hence a lower effective magnetization. Despite the modest *r_2_* values compared to other larger nanoparticle‐based systems, the *r_1_* values were more promising, yielding a *r_2_*/*r_1_* ratio of 4.2; enabling the use of the nanoparticles as *T_1_*‐*T_2_* dual‐mode contrast agents. Since *T_1_* relaxation largely depends on direct water contact, it is not surprising to see such an increase in *r_1_* when designing smaller‐sized magnetic nanoparticles with a larger surface area.^[^ ^81^ ^]^ Similarly, Uvdal and co‐workers have developed a dual *T_1_*‐*T_2_* contrast agent based on an SPN via a solvent‐mediated self‐assembly process. In this study, a solution of ultra‐small SPIONs (2.2 nm), a novel CP, and polystyrene‐b‐polyacrylic acid in tetrahydrofuran were treated slowly with water (20 wt.%), inducing aggregation with the aid of sonication to yield SPNs (*D_h_* = 20.3 nm). The resulting SPNs were shown to exhibit minimal cytotoxicity and a reasonably high *r_1_* value of 7.01 mM^−1^ s^−1^ and an *r_2_* value of 26.79 mM^−1^ s^−1^.^[^ ^107^ ^]^

The applications of SPNs doped with SPIONs have also been expanded to include probes for use in photothermal therapy (PTT),^[^ ^55^, ^56^, ^80^ ^]^ photodynamic therapy (PDT),^[^ ^108^ ^]^ photoacoustic imaging (PAI), and as agents for the controlled release of therapeutic drugs. For example, Liu and co‐workers utilized a low bandgap CP (PDPP3T) to co‐encapsulate SPIONs (7.5 nm) with an amphiphilic copolymer, mPEG2000‐DSPE via a nanoprecipitation reaction yielding SPNs with a *D_h_* of 165 nm.^[^ ^109^ ^]^ Interestingly, they reported an increase in the photoacoustic response on addition of the SPIONs, leading to extra heat generation and faster heat dissipation in the presence of SPIONs. In order to demonstrate the bimodal imaging capabilities, the authors measured the relaxivity performance, reporting an *r_2_* value of 98 mM^−1^ s^−1^, which is smaller than that reported for similar bimodal systems. To further improve the *r_2_* value, hybrid particles have been prepared consisting of highly ordered SPION clusters encapsulated by both a narrow bandgap CP (PCPDTBT) and a novel phospholipid using a phase transfer method.^[^ ^110^, ^111^ ^]^ Along with a remarkable *r_2_* relaxivity value of 309.3 mM^−1^ s^−1^ at 3 T, the clusters of Fe_3_O_4_ NPs inside the SPNs were able to enhance the photoacoustic effect 22‐fold compared to the SPN without Fe_3_O_4_ clusters. Results from transient absorption analysis suggest that the CP and SPIONs display a combined effect of light absorption and energy dissipation, which enhances the photoacoustic effect. The authors tentatively attributed the enhancement in both *T_2_* relaxation and photoacoustic effect on the structured ordering of clusters of SPIONs. Indeed, the ordering of SPIONs into clusters was shown to significantly increase the *r_2_* value. It is worth noting that there is some ambiguity in the calculation of the r_2_ value, as no information is provided on how the *R_2_* value varies as a function of iron concentration, which is commonly used to determine *r_2_*.

There is an optimal range in the size of the iron oxide clusters beyond which a decrease in relaxivity is observed. This was exploited in a hybrid system for the detection of cathepsin L, which is an endopeptidase that degrades proteins and plays various important roles in the body. The action of the enzyme degrades a poly(lysine) linker on the surface of the SPN and consequently promotes aggregation of SPIONs and SPNs via electrostatic interactions. The resulting formation of clusters over 200 nm in diameter led to a decrease in relaxivity (*r_2_*) indicating the presence of the cathepsin L.^[^ ^112^ ^]^

**Figure 11:**
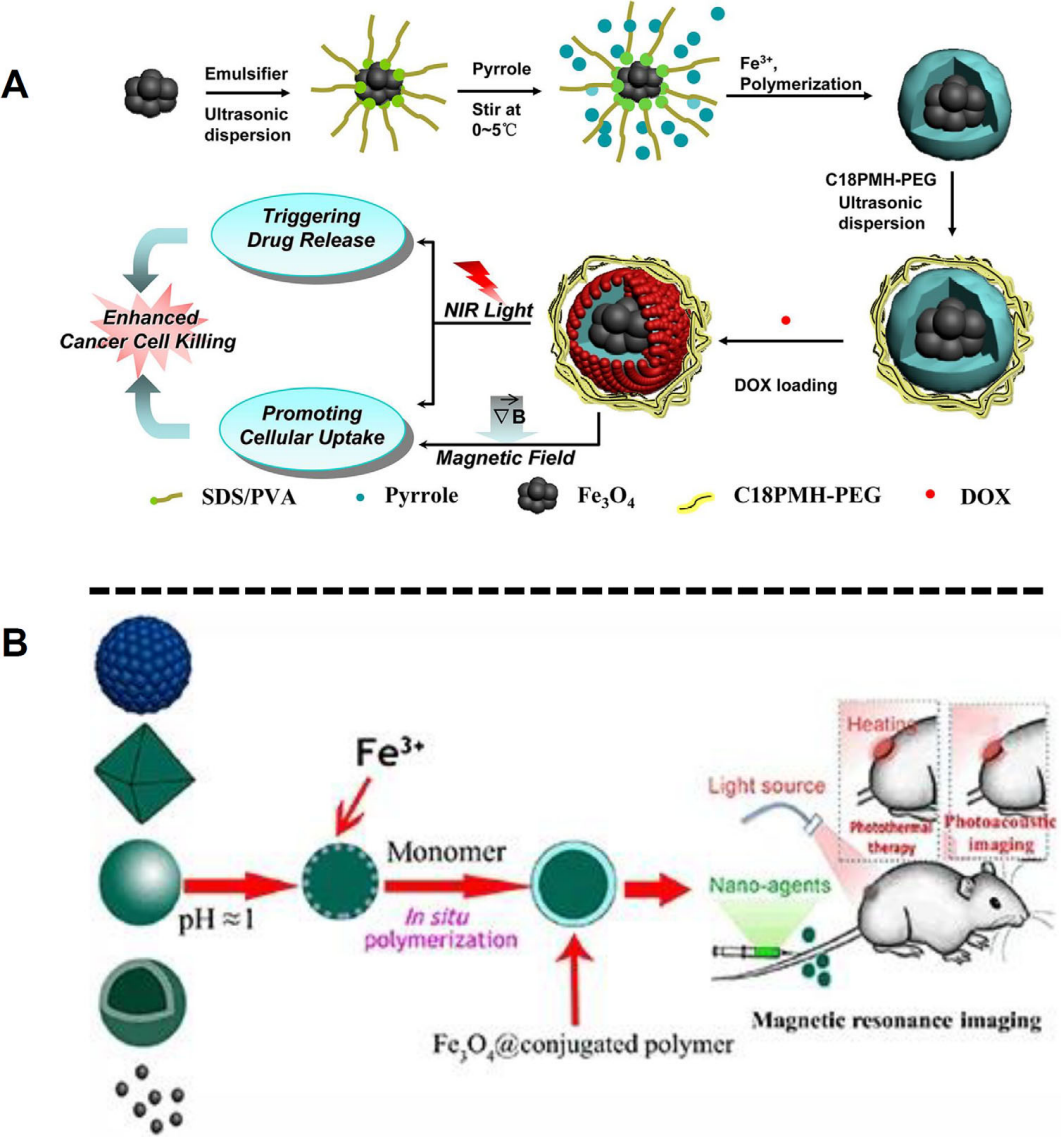
A) Illustration showing the preparation of SPION PPy‐PEG nanocomposites, their subsequent loading, and their use as stimuli response probes. Reproduced with permission.^[^ ^113^ ^]^ Copyright 2013, American Chemical Society. B) Schematic depicting the coating of SPIONs of various shapes with PPy performed at low pH. The resulting NPs can be used as theranostic probes. Reproduced (adapted) with permission.^[^ ^116^ ^]^ Copyright 2016, Tsinghua University Press and Springer‐Verlag Berlin Heidelberg.

The use of PPy NPs as probes for PAI and PTT has seen widespread use in recent years owing to the strong NIR absorption and excellent biocompatibility of these nanomaterials. A trimodal system consisting of an assembly of SPION clusters (8–10 nm) encapsulated within SPNs for use in *T_2_* imaging, fluorescence, and PAI has been reported. By performing an in situ chemical oxidation polymerization of pyrrole in the presence of SPIONs clusters, followed by coating with an amphiphilic PEG polymer, the authors prepared Fe_3_O_4_@PPy‐PEG core–shell nanoparticles (**Figure** **11**A). The resulting NPs displayed a *D_h_* of 150 nm and were subsequently shown to efficiently load and deliver doxorubicin (DOX). More importantly, the NPs exhibit a moderate *r_2_* value of 87 mM^−1^ s^−1^ at 3T. This modest increase in relaxivity may be attributed to the thick polymer layer on the surface and the use of PEG, which could adversely affect water accessibility, in addition to the small size of the NPs.^[^ ^113^ ^]^

Similar strategies have also been adopted to coat SPIONs with PPy for use as multimodal probes,^[^ ^114^, ^115^ ^]^ however, the preparation of a CP coating on the surface of the SPION is often performed under acidic conditions to slowly etch the SPION to generate Fe^3+^ on the surface, which catalyzes the polymerization (Figure 11B). This approach adversely affects the magnetization of the SPIONs and typically leads to a reduction in *r_2_* owing to the loss of Fe(II) ions. For example, Kang and co‐workers prepared a series of SPN‐coated SPIONs via an in situ polymerization reaction and found that there was a consistent reduction in the effective magnetization of the SPIONs from 90 emu g^−1^ for bare SPIONs to 79 emu g^−1^ for SPIONs coated with PPy.^[^ ^116^ ^]^

Interestingly, it was recently shown that PPy NPs can be modified to behave as *T_2_* contrast agents through the inclusion of polarons in the conjugated polymer backbone. Lin and co‐workers have prepared a series of PPy NPs containing catechol derivates and have demonstrated their ability to reduce the *T_2_* relaxation time of neighboring water molecules. The authors propose a mechanism whereby the catechol derivatizes promote spin‐spin interactions with neighboring water molecules encouraged by the presence of bipolarons and radical traps. Despite their ability to induce contrast, when compared to SPIONs, the PPy NPs deliver poor contrast, which the authors attribute to the small number of polarons found in the NPs.^[^ ^117^ ^]^

## 4 Conclusion and Future Perspectives

The use of semiconducting polymer nanoparticles (SPN) in the design of contrast agents (CA) for magnetic resonance imaging (MRI) has led to a variety of new functional materials (Table 1). In addition to the benefits of combining different modalities, this approach has the potential to address some of the shortcomings of conventional small molecule and monomodal MRI contrast agents. The superior photoluminescent and biological properties of SPNs make them an excellent platform for the incorporation of an additional imaging modality. In particular, the globular nature of the SPN significantly enhances the *r_1_* relaxivity of *T_1_*‐weighted contrast agents (e.g., based on Gd, Mn) whilst their excellent colloidal stability and ease of surface functionalization enables the incorporation of *T_2_*‐weighted contrast enhancement, such as from superparamagnetic iron oxide nanoparticles (SPIONs). A wide variety of design strategies have been adopted in the literature to develop SPN‐based CAs for MRI, although their efficacy in terms of their *r_1_* or *r_2_* relaxivity values is generally lower than that of other NP‐based CAs. This review summarises the key design strategies adopted by researchers in this field and highlights the advances made in improving relaxivity performance. However, despite the promising optical and MRI performance demonstrated by the reported systems listed in Table 1, only a few studies provide quantitative analysis of the enhancement effect compared to appropriate control groups. Most studies rely primarily on qualitative assessment of imaging results or visual comparisons of images, leading to general conclusions about performance improvement. The absence of standardized, control‐based quantitative data makes it challenging to objectively evaluate and compare the efficiency of various systems across different studies. To facilitate cross‐study comparisons and advance the field, future research should prioritize the inclusion of rigorous, comprehensive, and quantitative in vivo imaging performance analysis, such as signal‐to‐noise ratio (SNR), contrast‐to‐noise ratio (CNR), and other statistical metrics, using well‐defined control groups. Such data would greatly enhance the comparability of research outcomes in multimodal imaging.

In order to address the poor colloidal stability of surface functionalized SPNs, PEGylation has commonly been employed to improve the long‐term stability of these NPs under a wide range of conditions. Further, to enhance functionality, SPNs can be conjugated with biologically relevant ligands such as targeting peptides or antibodies, designed to enhance targeting and facilitate passage across biological barriers. However, ligand conjugation can influence properties such as water accessibility to paramagnetic centers, nanoparticle size, charge, and colloidal stability, potentially impacting relaxivity and optical characteristics.^[^ ^118^ ^]^ For example, the development of *T_1_*‐weighted CAs has been based either on the covalent attachment of a paramagnetic center to the end of a PEG chain, compromising the relaxivity gains from a reduction in the rotational correlation time, or buried within the PEG layer with reduced water accessibility. A more promising approach would be to directly attach the MRI contrast agent unit to the surface of the SPNs using a rigid linker. These ligand effects are generally more subtle and manageable compared with the pronounced impact of the thick polymeric SPN matrix itself. The type, density, and conjugation strategy of ligands should be carefully optimized to balance enhanced targeting with minimal adverse effects on imaging performance. However, systematic investigations of how surface ligands affect both relaxivity and fluorescence imaging characteristics remain limited, making this a key area for future research.

The nature of the paramagnetic center itself is also of importance as this affects both the relaxivity and the prospects for clinical translation of the CA. Indeed, there are established concerns over the use of Gd‐based CAs due to the potential for the release of free Gd^3+^ ions, which has been linked to nephrogenic systemic fibrosis (NSF) and, more recently, to the deposition of Gd in the brain.^[^ ^119^ ^]^ Relatively few attempts have been made to create Gd‐free SPN CAs and those that have been reported display a significant reduction in relaxivity compared to materials based on gadolinium, thus compromising their utility in MRI. The toxicity concerns surrounding the use of gadolinium have been addressed through the use of macrocyclic chelators (largely superseding acyclic designs), while enhancements in relaxivity per Gd unit allow lower loadings to be used. This has ensured that the majority of *T_1_*‐weighted CAs currently in clinical use and being developed are still based on this trivalent lanthanide metal. Where CAs have been reported to display exceptionally high *r_1_* values, further investigation is needed to unravel the parameters that are responsible for this performance enhancement. In addition to data recorded on clinical scanners, NMR dispersion (NMRD) profiles can be used to assess the relaxivity of contrast agents over a range of magnetic field strengths and allow fitting to theoretical models (e.g., Solomon‐Bloembergen‐Morgan theory) to obtain the factors that govern the relaxation effects observed. Of particular importance is an assessment of the outer sphere contribution to relaxivity from the bulk water molecules, which may account for some of the extremely high *r_1_* values reported in the literature. Additionally, the coupling between the local and global rotational times may also be assessed using this method which can be advantageous.

The combination of SPIONs and SPNs is often achieved by incorporating the SPION into the nanomaterial core, which usually results in a reduction in water accessibility, compromising relaxivity. This issue is further exacerbated by the use of an additional coating layer on the SPIONs to prevent fluorescence quenching, which can partially undermine the desirable magnetic properties. To circumvent this, the magnetization of the SPIONs can be increased either by creating larger crystals or through the use of clustering. However, an increase in the size of the SPIONs beyond a diameter of 20 nm typically results in a loss of superparamagnetism and therefore the latter option is usually preferred. In addition, a more compact hydrated surface layer may also improve the relaxivity of the CA in a similar fashion to *T_1_*‐weighted contrast agents.

The use of SPNs as multimodal agents for optical and magnetic resonance imaging holds significant promise but still faces several challenges. For instance, the relationship between the structure of SPNs and their optical properties, such as luminescence mechanisms and biexponential decay modes, is not fully understood, potentially limiting the optimization of SPNs for use in optical imaging. Moreover, the evaluation of the in vivo biocompatibility and toxicity of SPNs, including a greater focus on immune responses and long‐term toxicological effects, would benefit their adoption. An improved understanding of these aspects would aid the potential clinical translation of SPNs. To advance the field, interdisciplinary approaches combining materials science, chemical modification, and biomedical engineering will allow these challenges to be addressed, paving the way for the full potential of SPNs as multimodal imaging agents to be realized.

## Conflict of Interest

The authors declare no conflict of interest.
